# Co‐Dependency Revisited: An Integrative Review of Conceptualisations and Mental Health Outcomes

**DOI:** 10.1002/cpp.70265

**Published:** 2026-04-10

**Authors:** Elena Molina, Abigail Taiwo, Ben Grey

**Affiliations:** ^1^ School of Health, Medicine and Life Sciences University of Hertfordshire Hatfield UK; ^2^ South West London St Georges Merton CAMHS, Birches House, Birches Close, Cricket Green London UK

**Keywords:** attachment, co‐dependency, developmental trauma, integrative review, interpersonal dynamics, mental health

## Abstract

Co‐dependency is a contested construct, applied to a broad range of relational difficulties. Despite its relevance, the term remains conceptually fragmented. This limits research development and clinical recognition, hindering the creation of effective interventions. An integrative systematic review is therefore needed to consolidate recent evidence and clarify its clinical significance, aiming to synthesise conceptualisations and mental health outcomes. Following PRISMA 2020 guidelines and PROSPERO registration (CRD42024575573), Scopus, PubMed, ProQuest Central, ProQuest Dissertations & Theses Global, EBSCO, PsycINFO and PsycARTICLES were searched in September 2024 for peer‐reviewed studies published between 2013 and 2024. Eligible studies addressed co‐dependency's conceptualisation and/or mental health outcomes in adults. Risk of bias was assessed using MMAT and JBI for textual evidence. Thirty studies met inclusion criteria (14 quantitative, 9 qualitative and 7 theoretical). Narrative synthesis identified six conceptual perspectives: sociocultural, relational, addiction/pathology‐based, developmental, psychoanalytic and cognitive‐personality. Co‐dependency was consistently associated with emotional distress, disrupted identity and impaired relational functioning. Two integrative frameworks are proposed: one mapping intrapersonal and interpersonal contributors to co‐dependency, and one illustrating a perpetuating model of mental health outcomes. Limitations include limited cultural generalisability and the exclusion of studies addressing adjacent constructs. Strengths include methodological diversity, transparent quality appraisal and the generation of clinically relevant models. Findings support a shift towards relationally and developmentally informed understandings of co‐dependency.

## Summary

- Co‐dependency appears to reflect relational patterns shaped by developmental vulnerabilities, trauma and sociocultural expectations rather than individual pathology.
- Behaviours often labelled as pathological may function as adaptive coping strategies within earlier relational environments; clinicians should explore their meaning and function.
- Trauma‐ and attachment‐informed formulations can help identify how internal vulnerabilities intersect with recurring interpersonal patterns.
- Interventions should emphasise relational safety, boundary‐setting, emotional regulation and identity differentiation.
- Cultural context plays a role in shaping expectations of care, obligation and self‐sacrifice; practitioners should assess these influences explicitly.

## 1 Introduction

Co‐dependency is a contested construct (Bacon et al. 2020), initially rooted in addiction recovery literature but now applied to broader relational and psychological difficulties. It is commonly defined as a pattern of dependency on others at the expense of personal well‐being (Wright and Wright 1991), typically emerging in the context of concerned significant others (CSOs), that is family members or partners affected by a loved one's substance use disorder (SUD) or other caregiving relationships. Despite its relevance, co‐dependency lacks formal diagnostic criteria and remains conceptually ambiguous, with varied definitions (Hands and Dear 1994; Pagano‐Stalzer 2021).

Debates about the construct have spanned several perspectives, including sociocultural, relational, addiction/pathology, developmental, psychoanalytic and cognitive‐personality. Early models emerged from clinical observations in addiction‐affected families. Cermak (1986), emphasised self‐neglect and identity loss, noting that co‐dependency shared features with personality disorders (PDs), characterised by enduring patterns of thinking, feeling and relating that cause distress or impairment. Cermak's proposal that co‐dependency could warrant diagnostic recognition contributed to a lasting tendency to conceptualise it as individual pathology.

Relational and systemic theories extended these ideas, framing co‐dependency as a product of enmeshment and low self‐differentiation within families, where blurred boundaries perpetuate dependency patterns (Bowen 1978; Minuchin 1974). Psychoanalytic perspectives, from Horney's (1950) ‘morbid dependence’ to object relations theories, framed co‐dependency as a defensive adaptation marked by loss of self. In this light, co‐dependency was portrayed as more detrimental than alcoholism itself, with co‐dependents characterised as ‘volunteer‐victims’ who maintained the dysfunctional relationship to meet unmet needs (Troise 1993).

Subsequently, feminist critiques challenged this framing, arguing that it pathologised care and disproportionately targeted women, ignoring the sociocultural expectations that shape caregiving roles (Collins 1993). More recent perspectives have introduced developmental and attachment‐based frameworks, linking co‐dependency to early adversity and caregiving (Coffman and Swank 2021; Tunca et al. 2024), while cognitive‐personality frameworks highlight maladaptive traits and schemas (e.g., Bespalov et al. 2024). Neuropsychology research has also begun to expand on earlier pathology‐based framings, exploring potential biological correlates (e.g., Zielinski et al. 2019), although findings remain preliminary.

While these perspectives have expanded understanding of co‐dependency, they have largely developed in parallel rather than converging into a coherent conceptual framework. As a result, the field remains conceptually fragmented. Related constructs such as affective dependence (Sirvent‐Ruiz et al. 2022) and love addiction (Diotaiuti, Mancone, et al. 2022) further complicate co‐dependency's conceptual boundaries, hindering research synthesis and clinical application. While these constructs share overlap with co‐dependency (e.g., emotional reliance) and are sometimes used in place of the term, they are more often examined in romantic contexts, whereas co‐dependency has historically been applied to caregiving and addiction contexts. Affective dependence is often conceptualized as a distinct attachment‐based construct (Sirvent‐Ruiz et al. 2022), while love addiction has more frequently been discussed alongside co‐dependency along a continuum of maladaptive relational coping (Liverano et al. 2023).

Co‐dependency has been linked to mental health difficulties, including anxiety, depression, low self‐esteem and reduced life satisfaction (Happ et al. 2023; Knapek et al. 2021). These outcomes appear particularly prevalent among individuals with histories of relational trauma, such as CSOs, adult children of alcoholics (Cermak 1984) and survivors of abuse (Bornstein 2006). Prevalence estimates range from 10% to 20% in the general population to 36% in clinical groups (Hughes‐Hammer et al. 1998; Noriega et al. 2008).

Some recent reviews have examined aspects of co‐dependency, such as interventions (Abadi et al. 2015) or its impact on specific populations (Koudriavtseva 2025). However, these remain narrow in scope or outside the peer‐reviewed literature, and no systematic review has integrated both conceptual understanding and mental health outcomes. Many individuals self‐identify as co‐dependent and seek peer‐led support such as Co‐Dependents Anonymous (CoDA; Lancer 2015), highlighting the construct's continued relevance in clinical and lived‐experience contexts.

Given the absence of consensus definitions and the predominance of theoretical debate, an integrative review methodology was required to capture both empirical findings and conceptual developments. The present review aims to support psychological formulation and clinical understanding of co‐dependency as a relational coping pattern, rather than to establish it as a diagnostic category. This review addresses two questions:

1. How has co‐dependency been conceptualised in the past decade?
2. What mental health outcomes are associated with co‐dependency?

Together, these questions aim to generate an integrative understanding of co‐dependency that advances conceptual clarity and informs clinical application.

## 2 Method

The systematic literature review followed PRISMA guidelines (Page et al. 2021) and an integrative methodology. Given co‐dependency's conceptual ambiguity, an integrative review was needed to incorporate both empirical and theoretical work. Much of the construct's development has occurred through conceptual debate, and empirical studies alone could not capture this breadth. The review was preregistered with PROSPERO (ID: CRD42024575573; https://www.crd.york.ac.uk/PROSPERO/view/CRD42024575573).

During full‐text screening, two amendments were made to refine the scope and methodology. First, although no time limit was initially applied to capture the historical evolution of the construct, the inclusion criteria were narrowed to studies published from January 2013 onward, ensuring contemporary relevance and reducing overlap. Second, because of the heterogeneity of study designs and outcomes, a narrative synthesis approach was adopted in place of the originally planned thematic analysis (TA). Narrative synthesis was considered more appropriate for integrating conceptual and empirical literature, as it allowed theoretical papers to inform the development and structuring of themes, while empirical studies were used to illustrate, test or elaborate these conceptual positions, supporting an interpretative synthesis of a complex and heterogeneous evidence base (Popay et al. 2006).

Although grey literature was initially included in the search strategy, only peer‐reviewed studies were retained for final synthesis, given the volume and quality of eligible evidence. While grey literature can add complementary and contextual evidence (Paez 2017), it was considered not essential where peer‐reviewed sources were judged as providing sufficient conceptual coverage (Adams et al. 2017). These changes were made prior to final data extraction and are transparently reported here.

### 2.1 Researcher Positionality

Given the interpretive nature of this review, the researchers' positionality is acknowledged. As psychologists with a focus on relational dynamics, the authors approached the synthesis with sensitivity to issues of attachment and trauma. Personal and professional experiences may have informed the view of co‐dependency as a complex, potentially adaptive construct. Here, ‘adaptive’ refers to strategies that were functional within an individual's developmental or sociocultural environment, even if they later become maladaptive in adult relationships. Reflexivity and transparency were maintained throughout study selection and interpretation.

### 2.2 Search Strategy

A scoping search (August 2024) was followed by a fully systematic search, completed in September 2024 across Scopus, PubMed, ProQuest Central, ProQuest Dissertations & Theses Global, EBSCO, PsycINFO and PsycARTICLES. Reference lists of the included studies were also screened. Search terms targeted the following three domains: (1) co‐dependency and related constructs, (2) conceptual frameworks and (3) mental health outcomes. Full search strategies, including database‐specific strings and filters, are provided in the Supplementary Material (see Supplementary File 1).

### 2.3 Eligibility Criteria

Inclusion and exclusion criteria were developed using the SPIDER framework (Sample, Phenomenon of Interest, Design, Evaluation, Research type; Cooke et al. 2012). This approach was selected to accommodate both empirical and theoretical studies, in line with the review's integrative aims. Table 1 outlines the full SPIDER criteria.

**TABLE 1:** SPIDER framework.

SPIDER	Inclusion criteria	Exclusion criteria
**S**ample	Published studies involving empirical data or theoretical discussion of co‐dependency in adult populations.	Studies focusing on children and adolescents.
**P**henomenon of **I**nterest	Conceptualisation of co‐dependency and associated mental health outcomes	Studies using related terms without explicit mention of co‐dependency. Studies providing little theoretical contribution
**D**esign	Empirical or theoretical studies published in peer‐reviewed journals or scholarly outlets. Grey literature, expert opinions or commentaries were eligible if offering substantial theoretical contribution. Published after January 2013.	Single or multiple case studies. Grey literature, expert opinions or commentaries lacking significant theoretical contributions. Studies published before January 2013.
**E**valuation	Outcomes related to co‐dependency conceptualisation and mental health (e.g., anxiety, depression, stress, well‐being)	Studies focusing primarily on SU or intervention without broader focus on co‐dependency.
**R**esearch Type	Quantitative, qualitative, mixed methods, theoretical papers/commentaries	Non‐English language studies; studies without full‐text availability.

### 2.4 Selection Process

Covidence and Zotero were used to manage records. Two reviewers independently screened titles, abstracts and full texts. Discrepancies, mostly around conceptual contribution, were resolved through discussion. Interrater agreement was substantial (Cohen's *κ* = 0.69 at abstract screening; *κ* = 0.65 at full‐text; Park and Kim 2015).

### 2.5 Data Extraction

One reviewer independently extracted data from all included studies using standardised extraction templates. No automation tools were employed. Extracted data encompassed study characteristics (participant demographics, study aims, design and methodology, country of origin and publication year) as well as definitional framing, theoretical frameworks and key findings/arguments related to conceptualisations and/or mental health outcomes, including anxiety, depression, stress, well‐being, emotional distress and related psychological indicators. To enhance reliability, ambiguous cases were discussed within the research team. Where participant details were missing or unclear, no assumptions were made; such data were recorded as ‘not reported.’ No attempts were made to contact study authors for further information.

### 2.6 Quality Appraisal

Quality appraisal was carried out independently by the first author. The Mixed Methods Appraisal Tool (MMAT; Hong et al. 2018) was applied to empirical studies to ensure consistency across designs and avoid the bias associated with using multiple tools (Pati and Lorusso 2018). The MMAT includes criteria tailored to qualitative, quantitative and mixed‐methods research, focusing on aspects such as the clarity of research questions, appropriateness of methodology and transparency of data collection and analysis. For nonempirical papers, the JBI Critical Appraisal Checklist for Textual Evidence (McArthur et al. 2015) was used, selected for its rigour and recent updates. This tool is designed for theoretical or opinion papers, evaluating whether arguments are well‐grounded, consistent and supported by evidence.

Neither tool generates formal quality ratings (e.g., high/moderate/low); instead, studies were appraised against domain‐specific criteria and judged as sufficiently rigorous for inclusion based on transparency, methodological coherence and relevance. A study was judged sufficiently rigorous if it met most criteria relevant to its design; those with more than two ‘No’ responses on the appraisal checklists were considered lower quality but still included if findings were transparently reported.

### 2.7 Data Synthesis Method

The synthesis was guided by Whittemore and Knafl's (2005) framework, which is well‐suited for combining diverse study designs and data types to develop a comprehensive understanding of co‐dependency. Studies were grouped according to their primary focus: Those reporting outcome measures were synthesised to address the second review question (mental health outcomes), while all other studies were synthesised to address the first review question (conceptualisations). Studies were allocated to groups based on study type, conceptualisations and mental health outcomes reported. Within each group, findings were thematically organised and narratively analysed based on the type of conceptualisation or reported outcome.

Theme development was primarily deductive, guided by the review questions and existing literature, but refined inductively through constant comparison to capture both convergences and divergences across studies. Data extraction and display tables provided traceability of study contributions, supporting transparency.

Conceptual and empirical studies were integrated in complementary ways: Conceptual papers introduced debates and theoretical shifts, while empirical studies illustrated or tested these perspectives with observed data. Qualitative studies were treated as equally valuable to quantitative work. To minimise subjectivity, patterns were cross‐checked across study types, themes were aligned closely with the review questions, and weaker quality studies were weighted cautiously in shaping conclusions.

### 2.8 Confidence Assessment Method

A confidence assessment was conducted for each key review finding and is presented following the results. Given the integrative nature of the review, formal tools were not appropriate. Instead, a narrative confidence assessment was applied, drawing on four key criteria: methodological quality (assessed via MMAT and JBI checklists), consistency (replication across studies, methods or contexts), relevance (direct focus on co‐dependency) and data richness (depth of reporting, e.g., detailed qualitative accounts or robust quantitative analyses).

Overall ratings were assigned using a threshold approach. Findings were rated high when most contributing studies were methodologically robust and scored strongly on at least three of the four criteria; moderate when evidence was mixed or uneven; and low when based on few, lower‐quality or inconsistent studies. Confidence levels therefore reflected the collective strength and coherence of the contributing evidence.

## 3 Results

### 3.1 Study Selection

**FIGURE 1:**
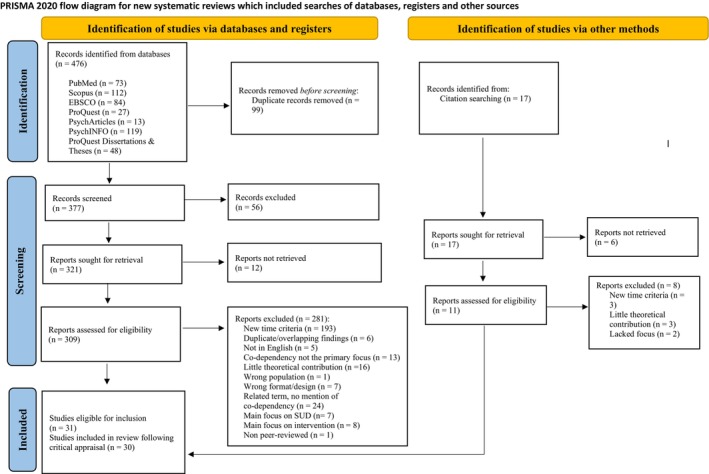
PRISMA flowchart.

A PRISMA flowchart (Figure 1) summarises the search conducted in August–September 2024. The search yielded 476 records from databases and 17 from citation searching. After removing duplicates, 377 records were screened by title and abstract, with 56 excluded. Of these, 321 full‐text reports (from databases) were sought for retrieval, with 12 not retrievable, leaving 309 assessed for eligibility. From the citation search, six records were not retrievable and 11 were assessed in parallel at the full‐text stage. A list of excluded full‐text articles and reasons for exclusion can be seen in Supplementary File 2 (Table B1). A total of 31 studies were deemed eligible for inclusion, and following quality appraisal, 30 were retained for synthesis.

### 3.2 Study Characteristics

The review included 30 studies: 17 quantitative, five qualitative and eight conceptual. Of the conceptual works, one incorporated qualitative elements, one was a review, and six were theoretical or expert opinion pieces. Tables 2, 3, 4 summarise the characteristics by study type, with full details in Supplementary File 3 (Tables C1–C3).

**TABLE 2:** Summary characteristics of quantitative studies.

**Atintas and Tutarel‐Kislak** **2019** ***Turkey***	
Type	Correlational and comparative
Population	Wives of alcoholics and a comparison group (*N* = 100).
Method	Purposive sampling (clinical group); convenience sampling (comparison). Validated questionnaires. Analysed using *t*‐tests and regression.
Co‐dependency definition	A pattern of other‐focus and self‐neglect involving low self‐worth, concealment of self, psychosomatic symptoms and unresolved family‐of‐origin issues.
Findings	Conceptualisation: Co‐dependency is higher in wives of alcoholics; mental health outcomes: Co‐dependency negatively correlated with marital adjustment, power and life satisfaction as well as positively correlated with depression, anxiety and stress.
**Bespalov et al.** **2024** ***Turkey***	
Type	Correlational and comparative
Population	Co‐dependent couples (*N* = 85)
Method	Random purposive sampling. Validated questionnaires. Analysed using *t*‐tests, ANOVA and correlation.
Co‐dependency definition	A socio‐psychological phenomenon involving emotional interdependence, low self‐worth and autonomy, particularly in the context of marital relationships.
Findings	Conceptualisation: Co‐dependency linked to low self‐esteem and emotional intelligence. Women showed higher empathy and emotional reactivity; men coped through confrontation and distancing.
**Chang** **2018** ***Taiwan***	
Type	Correlational
Population	College students (*N* = 576)
Method	Convenience sampling. Validated questionnaire. Analysed using correlation and structural equation modelling (SEM).
Co‐dependency definition	A complex construct characterised by low self‐differentiation, other‐focused behaviours and relationship anxiety, emerging from family‐of‐origin dysfunction.
Findings	Conceptualisation: Co‐dependency was associated with social dysfunction and poor psychological adjustment. Lower self‐differentiation partially mediated the link between family‐of‐origin dysfunction and co‐dependency.
**Ehsan and Suneel** 2020 ***Pakistan***	
Type	Correlational
Population	Parents with intellectually disabled children (*N* = 41).
Method	Convenience sampling. Validated questionnaires (SF‐CDS, DASS‐21). Analysed using correlation and ANCOVA.
Co‐dependency definition	Dysfunctional ways of relating, can develop in response to caregiving burden.
Findings	Mental health outcomes: Co‐dependency negatively correlated with mental health functioning. Gender did not significantly predict mental health when co‐dependency was controlled for.
**EvgIn and Sümen** 2022 ***Turkey***	
Type	Descriptive and correlational
Population	Health care students (*N* = 292).
Method	Convenience sampling. Validated questionnaires. Analysed using Mann–Whitney *U*, Kruskal–Wallis and correlation.
Co‐dependency definition	A psychosocial pattern involving maladaptive coping, impaired self‐worth and relational dysfunction, shaped by early attachment experiences and sustained through adult caregiving roles.
Findings	Conceptualisation: There is a positive relationship between childhood neglect and abuse and co‐dependency. Mental health outcomes: A negative relationship was found between co‐dependency and self‐esteem, depression and stress‐coping.
**Happ et al.** **2023** ***Hungary***	
Type	Correlational
Population	Adults in a relationship (*N* = 246)
Method	Convenience sampling; validated questionnaires. Analysed using correlation and SEM.
Co‐dependency definition	A stable attitude that determines a person's perception and behaviour, manifesting in dysfunctional pattern of relating to others.
Findings	Conceptualisation: Co‐dependency is associated with negative dyadic coping and perception of relationship problems. Mental health outcomes: Co‐dependency, negative dyadic coping and relationship problems perception predicted lower life satisfaction.
**Hawkins and Hawkins** **2014** ***USA***	
Type	Correlational and comparative
Population	Social work undergraduates (*N* = 208).
Method	Convenience sampling. Validated questionnaires. Analysed using MANOVA, ANOVA, regression.
Co‐dependency definition	A dimension of personality, varying by degree from normality to deviance, as operationalised by gender‐stereotyped attributes.
Findings	Conceptualisation: Co‐dependence does not differ by gender and is more prevalent among students with a positive family history of alcohol problems. It is negatively correlated with socially desirable masculinity and femininity traits and is linked to shame and vulnerability to depression. Contradependence is associated with sensation seeking, negative masculinity and problem drinking tendencies.
**Kaplan** **2023** ***Turkey***	
Type	Correlational and descriptive
Population	Housewives (*N* = 371).
Method	Snowballing sampling; validated questionnaires. Analysed using observed variable path analysis, SEM.
Co‐dependency definition	A characteristic that develops in dysfunctional families, associated with self‐neglect, other‐focus and suppression.
Findings	Mental health outcomes: mental health correlated with co‐dependency and self‐perception. Higher co‐dependency and negative self‐perception increased the psychological symptoms.
**Karasar** 2020 ***Turkey***	
Type	Correlational
Population	Preteachers (*N* = 188).
Method	Convenience sampling. Validated questionnaires. Analysed using SEM.
Co‐dependency definition	A pattern of self‐neglect, driven by social approval need, perfectionism and cultural expectations of self‐sacrifice, leading individuals to prioritise others' needs over their own.
Findings	Conceptualisation: Social approval plays a partial mediating role in the relationship between perfectionism and co‐dependency, suggesting that the need for social validation is a key factor linking perfectionistic tendencies to co‐dependency.
**Kaya et al.** **2024** ***Turkey***	
Type	Correlational
Population	Adults in different life stages (*N* = 401).
Method	Convenience sampling; validated questionnaires. Analysed using regression.
Co‐dependency definition	Relationship addiction: a pathological condition characterised by overreliance on interpersonal relationships.
Findings	Childhood emotional abuse and neglect contribute to co‐dependency. Resilience partially mediates the relationship between abuse and co‐dependency but not neglect.
**Knapek et al.** **2017** ***Hungary***	
Type	Comparative
Population	Adults engaging with psychiatry, self‐help groups or from the general population (*N* = 407).
Method	Convenience sampling; a validated questionnaire and a clinical interview. Analysed using chi‐square tests.
Co‐dependency definition	A mental problem characterised by extreme caretaking, enabling behaviour and responsibility for others.
Findings	Borderline and dependent traits are common among co‐dependents. However, 16% of co‐dependents do not display these traits, suggesting that co‐dependency can exist as a distinct concept separate from PD.
**Knapek et al.** **2021** ***Hungary***	
Type	Predictive
Population	Hungarian adults engaging with psychiatry, self‐help groups or from the general population (*N* = 192).
Method	Convenience sampling. Validated questionnaires and a clinical interview. Analysed regression.
Co‐dependency definition	A behavioural addiction, which can play a role in maintaining others' addictive behaviours.
Findings	Co‐dependency is predicted by subjugation and self‐sacrifice schemas, mental disorder diagnosis, female gender, borderline traits and parentification.
**Lampis et al.** **2017** ***Italy***	
Type	Correlational/predictive
Population	Adults in a relationship (*N* = 318).
Method	Convenience sampling; validated questionnaires. Analysed using *t*‐test and regression.
Co‐dependency definition	An affective disorder developing from the internalisation of family‐of‐origin experiences. It manifests as a relationship addiction.
Findings	The dimensions of self‐differentiation were more important in explaining co‐dependency compared to dyadic adjustment. The most important variables in predicting co‐dependency were emotional reactivity and emotional cut‐off.
**Rozhnova et al.** **2020** ***Russia***	
Type	Case–control
Study aim	To study the psychological and genetic components of co‐dependency.
Population	Three women groups: (1) co‐dependents, (2) phenotypically healthy; and (3) general population (*N* = 256).
Method	Convenience sampling; a validated questionnaire, a clinical interview, a projective test, a screening tool and a validated scale. Analysed using ANOVA, *t*‐test, chi‐square.
Co‐dependency definition	An addictive behaviour disorder influenced by early family dynamics, unmet needs and dysfunctional relationships.
Findings	Conceptualisation: Codependency has psychological and genetic components. Co‐dependent women showed auto aggressive behaviours and a family history of alcoholism. Mental health outcomes: risk of mental and physical health issues, psycho‐emotional overstrain, somatoform disorders.
**Tunca et al.** **2024** ***Turkey***	
Type	Correlational and comparative
Population	A SUD group and a control group (*N* = 115).
Method	Purposive sampling. Validated questionnaires. Analysed using *t*‐tests, correlations and regression.
Co‐dependency definition	A condition involving psychopathology, dysfunctional family systems and maladaptive relational patterns. It manifests as self‐neglect, low self‐worth and preoccupied attachment, with an overemphasis on others.
Findings	Conceptualisation: Codependency is influenced by defences, family dysfunction and preoccupied attachment.
**Vederhus et al.** **2019** ***Norway***	
Type	Validation and correlational study
Population	CSO of SUD patients and a control group (*N* = 664).
Method	Convenience sampling; validated questionnaires. Analysed using confirmatory factor analysis and regression.
Co‐dependency definition	A phenomenon characterised by self‐sacrifice, interpersonal control and emotional suppression.
Findings	Conceptualisation: COS of individuals with SUD exhibit higher co‐dependency, characterised by greater emotional suppression and interpersonal control. Mental health outcomes: Co‐dependency was associated with greater family dysfunction and worse quality of life.
**Zielinski et al.** **2019** ***USA***	
Type	Quasi‐experimental with correlational and comparative elements.
Population	CSO of individuals with SUD and a control group (*N* = 38).
Method	Sampling: purposive; data collection: Experimental task with image stimuli and a validated questionnaire. Analysed using functional near‐infrared spectroscopy processing, *t*‐test and correlations.
Co‐dependency definition	A learned dysfunctional condition, manifesting as excessive focus on a loved‐one with SUD despite negative consequences.
Findings	Conceptualisation: Co‐dependency is negatively associated with left dorsomedial prefrontal cortex activation in response to images of a loved one with SUD. Mental health outcomes: Brain activation suggests that co‐dependency may impair the ability to effectively regulate emotions in response to relationship stress.

**TABLE 3:** Summary characteristics of qualitative studies.

**Aristizábal** 2020 ***Columbia***	
Population	Imprisoned women reporting a romantic bond (*N* = 27).
Method	Purposive sampling. In‐depth interviews, focus groups and a validated scale. Analysed using descriptive and discourse analysis.
Co‐dependency definition	Emotional dependency characterised by a cycle of control and enabling behaviours.
Findings	Conceptualisation: Co‐dependency is influenced by gender roles, leading to denial, incomplete identity, repression and rescuing; mental health outcomes: Co‐dependency contributed to crime involvement, distress and relationship challenges as reflected in themes: (1) I did it for him, (2) Although he does not love me and (3) I preferred to remain silent.
**Bacon et al.** **2020** ***UK***	
Population	CoDA fellows (*N* = 8).
Method	Purposive sampling. In‐depth semi structured interviews and a visual method. Analysed using IPA.
Co‐dependency definition	A complex, psychosocial problem, seen as both an adaptive coping strategy and a socially accepted form of addiction.
Findings	Conceptualisation: Participant conceptualised co‐dependency as manifesting through emotional instability and an unclear sense of self and resulting from difficult childhood experiences.
**Klimczak and Klejna** **2018** ***Poland***	
Population	Co‐dependent women receiving psychological support (N = 32).
Method	Purposive sampling. Semistructured narrative interviews, with timeline drawing method. Analysed using narrative and thematic content analysis applying the Big five.
Co‐dependency definition	Adaptive response to stress and relational trauma, particularly in dysfunctional family settings.
Findings	Conceptualisation: Co‐dependent behaviours are a manifestation of childhood trauma. Co‐dependency is associated to high levels of neuroticism and conscientiousness, a moderate level of agreeableness and low levels of openness to experiences and extroversion.
**Nordgren et al.** **2020** ***Sweden***	
Population	Parents of adult children with SUD (*N* = 32).
Method	Purposive sampling. Semistructured interviews. Analysed using TA.
Co‐dependency definition	A range of behaviours shaped by societal expectations among individuals who are affected by SUD of family members.
Findings	Conceptualisation: Co‐dependency appears to be more of an externally attributed label than an internally recognised identity, at least initially. Mental health outcomes: Participants faced distress due to co‐dependency, experiencing guilt and ambivalence between supporting their children and setting boundaries, as a response to reconcile societal expectations.
**Sobol‐Goldberg et al.** **2024** ***Israel***	
Population	Women CSO of individuals in treatment for SUD (*N* = 12).
Method	Purposive sampling. Semistructured interviews. Analysed using content analysis.
Co‐dependency definition	A relational phenomenon that has negative connotations and may affect the way people and society relate to family members of individuals with addiction.
Findings	Mental health outcomes: Women internalised three types of social messages, which impacted on their wellbeing: (1) Messages leading to guilt, shame and self‐stigma, (2) messages contributing to exclusion and isolation and (3) messages supporting their caregiving role, which sometimes strengthened their sense of value.

**TABLE 4:** Summary characteristics of conceptual studies.

**Bacon and Conway** **2023** ***UK***	
Type	Commentary
Method	Literature Review & Case Illustration
Co‐dependency definition	A complex condition rooted in early family dynamics and unmet needs and involving maladaptive schemas.
Framework	Schema therapy; family system theory.
Key Argument	Co‐dependency is an outward manifestation of enmeshment, characterised by impaired autonomy and self‐sacrifice.
**Calderwood and Rajesparam** **2014** ***Canada***	
Type	Commentary
Method	Critical analysis
Co‐dependency definition	A stigmatising term describing COS as having dysfunctional traits.
Framework	Stress‐coping perspective.
Key Arguments	There is no evidence that the co‐dependency concept can be successfully applied to problem gambling. This application is problematic due to the stigma. The stress‐coping model is proposed as a more empowering perspective, framing these behaviours as adaptive strategies.
**Coffman and Swank** ***USA***	
Type	Theoretical paper
Method	Narrative synthesis of existing literature.
Co‐dependency definition	A dysfunctional learned behaviour pattern influenced by insecure attachment styles.
Framework	Attachment theory; family system theory.
Key Arguments	Insecure attachment styles are predictors of poor emotion regulation and interpersonal communication problems, which in turn may lead to co‐dependent behaviours. SUD in families significantly impacts attachment systems, leading to increased co‐dependency.
**Kolenova et al.** **2023** ***Russia***	
Type	Theoretical paper.
Population	N/A
Method	Theoretical review.
Co‐dependency definition	A nonchemical addiction caused by a change in value‐semantic constructs and a lack of necessary competencies, formed under the influence of negative experience of dysfunctional relationships.
Framework	Biopsychosocial perspective; cognitive/personality psychology.
Key arguments	Psychological markers of co‐dependent behaviour are manifested through a learned set of behavioural patterns, adaptation disorders and associations with various PD. Co‐dependency is associated to anxiety, depression and stress and has high comorbidity with PDs.
**Liverano et al.** ***Italy***	
Type	Theoretical paper
Method	Literature integration and development of a treatment protocol based on transactional analysis (TA)
Co‐dependency definition	The most common type of love addiction.
Framework	Attachment theory; psychodynamic theory; transactional analysis model.
Key Arguments	Co‐dependency is characterised by low self‐esteem, insecurity and a need to hold onto a partner to fulfil unmet needs. Co‐dependents tolerate mistreatment and assume a caregiver role.
**Shishkova and Bocharov** ***Russia***	
Type	Theoretical paper
Method	Theoretical review
Co‐dependency definition	A phenomenon rooted in the stigma associated with traditional female roles in families dealing with addiction. The behaviours traditionally associated with co‐dependency are instead conceptualised as a result of stress and burnout
Framework	Sociocultural perspective; burnout/stress‐coping perspective.
Key arguments	Co‐dependency frames caregiving behaviours as dysfunctional. The stress‐coping model can be employed instead to emphasise adaptive strategies.
**Weiss** **2019** ***USA***	
Type	Expert opinion with theoretical and quantitative elements
Population	Clinicians treating loved ones of sex addict (*N* = 64).
Method	Purposive sampling. Informal clinician survey (pre/posteducational session) to support conceptual model (prodependence). Analysed using descriptive survey analysis
Co‐dependency definition	A deficit‐based, trauma‐informed model, where caring for others is seen as dysfunctional behaviour.
Framework	Attachment theory; stress‐coping perspective.
Findings	The co‐dependency model implies that COS are dysfunctional. In contrast, Prodependence is introduced and welcomed as an alternative strength‐based, attachment‐focused model. This model frames COS behaviour as normative and rational responses to a relational crisis.
**Winter** **2019** ***Sweden***	
Type	Theoretical with qualitative elements
Population	Participants in the Forum for Research on Drug Dependence network events in Sweden.
Method	Observations, website materials and field notes from a public meeting. Analysed using content analysis.
Co‐dependency definition	A construct shaped by societal narratives of victimisation and the brain disease model.
Framework	Social constructionism
Findings	Co‐dependency knowledge was shaped by victimisation narratives and the biological model of addiction. Professionals may have had an agenda to promote the biological model to ensure co‐dependency aligns with scientific authority.

Studies were conducted across 15 countries, most frequently Turkey (*N* = 6), the USA (*N* = 4), Hungary (*N* = 3) and Russia (*N* = 3). Sample sizes ranged from 38 (Zielinski et al. 2019) to 664 (Vederhus et al. 2019) for quantitative studies, and 8 (Bacon et al. 2020) to 32 (Klimczak and Klejna 2018; Nordgren et al. 2020) for qualitative. Most participants were female (76%), aged 18–81 years (M = 35.5).

Quantitative data were primarily collected via surveys (e.g., Karasar 2020; Knapek et al. 2021) including self‐report instruments such as the Spann–Fischer Codependency Scale (e.g., Happ et al. 2023), the Codependency Assessment Tool (e.g., EvgIn and Sümen 2022), and measures of attachment, well‐being and mental health outcomes. Some studies used clinical interviews (e.g., IC10 Clinical interview in Rozhnova et al. 2020) or quasi‐experimental methods (e.g., neuroimaging in Zielinski et al. 2019). Qualitative studies employed interviews (e.g., Nordgren et al. 2020), focus groups (Aristizábal 2020) and creative methods such as object‐based elicitation (e.g., Bacon et al. 2020) and timeline drawing (e.g., Klimczak and Klejna 2018).

Statistical approaches included correlation and regression (e.g., Happ et al. 2023) and group comparison (e.g., Bespalov et al. 2024). Qualitative analysis varied, including TA (e.g., Nordgren et al. 2020), discourse analysis (Aristizábal 2020) and content analysis (Sobol‐Goldberg et al. 2024).

Study contexts included CSO (e.g., Atintaş and Tutarel‐Kışlak, 2019), caregiving (e.g., Ehsan and Suneel 2020), recovery groups (e.g., Bacon et al. 2020) and general relationships (e.g., Lampis et al. 2017). Theoretical frameworks spanned family systems (e.g., Chang 2018), attachment (e.g., Weiss 2019) personality (e.g., Hawkins and Hawkins 2014), psychodynamic (e.g., Tunca et al. 2024) social psychology (e.g., Karasar 2020) and trauma perspectives (e.g., Kaya et al. 2024). Mental health outcomes were reported in 11 studies (e.g., Happ et al. 2023; Knapek et al. 2021).

### 3.3 Quality Appraisal

A summary of appraisal outcomes is provided in the Supplementary File 4 (Tables D1–D3). One study (Kolenova and Kukulyar 2024) was excluded following retraction, leaving 30 studies for synthesis. Overall, these were judged as being of sufficient quality to inform the review.

Most studies had clearly defined aims and appropriate methodologies, with coherent interpretations. One qualitative study (Klimczak and Klejna 2018) lacked transparency in how findings were derived. Other limitations included small or site‐specific samples (e.g., Happ et al. 2023), which were common across studies. Reliance on nonrepresentative samples, while common in correlational research, constrains generalisability; findings should therefore be interpreted with appropriate caution.

While interviews were the primary method in qualitative studies, some used innovative approaches like object‐based elicitation (e.g., Bacon et al. 2020). Reflexivity was inconsistently addressed, some studies omitted researcher positioning, while others (e.g., Bacon et al. 2020) employed peer‐debriefing and journaling.

Conceptual papers were generally robust, though a few lacked referencing (e.g., Weiss 2019) or critical engagement with opposing views (e.g., Winter 2019). Overall, findings were clearly presented, theoretically grounded and deemed sufficiently robust to contribute to synthesis.

Quality assessments informed how evidence was weighted in the synthesis: Studies with weaker transparency or small, site‐specific samples (e.g., Ehsan and Suneel 2020; Klimczak and Klejna 2018) were treated cautiously and did not drive conclusions, while methodologically stronger studies carried greater weight in shaping themes. These appraisals, together with evaluation of consistency, relevance and data richness, also contributed to the certainty assessment applied to review findings (see Supplementary File 6, Table F1).

### 3.4 Narrative Synthesis

#### 3.4.1 The Evolving Debate of Co‐Dependency

Six overarching themes and their related subthemes were identified during the synthesis (Table 5). These themes were developed through an iterative, interpretive process informed by the review questions and refined through constant comparison across qualitative, quantitative and conceptual studies. In addressing the first research question (How has co‐dependency been conceptualised over the past decade?), six conceptual perspectives were identified. Many studies contributed to more than one category, reflecting the conceptual overlap that characterises this field. Detailed study‐to‐theme mapping is provided in Supplementary File 5, Table E1.

**TABLE 5:** Narrative themes and subthemes.

Themes	Subthemes
Theme 1: The evolving debate of co‐dependency	‐Sociocultural perspectives ‐Relational perspectives ‐Addiction and pathology perspectives ‐Developmental perspectives ‐Psychoanalytic perspectives ‐Cognitive‐personality perspectives
Theme 2: The impact of co‐dependency on wellbeing	‐Emotional and psychological wellbeing ‐Self‐concept and identity ‐Relational and social functioning

#### 3.4.2 Sociocultural Perspectives

This conceptualisation frames co‐dependency as shaped by gendered socialisation, cultural norms and institutional narratives. Hogg and Frank (1992) introduced co‐dependence and contradependence as opposing relational styles shaped by early experience and gender norms. Hawkins and Hawkins (2014) developed this further, positioning these constructs at opposite ends of an agency–communion continuum. Rather than focusing on biological sex, they argued these patterns arise from socially constructed gender roles. Gendered expressions of co‐dependency were also supported by Bespalov et al. (2024).

Several studies linked co‐dependency to patriarchal dynamics. Aristizábal (2020) and Kaplan (2023) described caregiving as internalised identity in women shaped by societal expectations. Atintas and Tutarel‐Kislak (2019) identified power imbalances reinforcing women's emotional labour, while Sobol‐Goldberg et al. (2024) highlighted internalised stigma around co‐dependency despite cultural ideals of selflessness.

Cultural frameworks also informed this conceptualisation. Karasar (2020) and EvgIn and Sümen (2022) associated Turkish collectivism with overfunctioning, while Lampis et al. (2017) described conflicting cultural values in Italy shaping relational boundaries. Finally, social constructionist work (Nordgren et al. 2020; Winter 2019) challenged pathologising discourses, viewing co‐dependency as a label assigned through societal and institutional processes rather than inherent pathology.

#### 3.4.3 Relational Perspectives

This conceptualisation situates co‐dependency within interpersonal and systemic dynamics. Systemic theory was central, with co‐dependency commonly linked to low self‐differentiation (Bowen 1978) and enmeshment (Minuchin 1974). Chang (2018) and Lampis et al. (2017) identified low differentiation as a key predictor, with Chang further demonstrating its mediating role between family dysfunction and co‐dependency. Qualitative work similarly described diminished differentiation within close relationships (Bacon et al. 2020), while Bacon and Conway (2023) framed co‐dependency as a function of enmeshment.

Building on these dynamics, family functioning models highlighted relational dysfunction across both addiction‐affected and nonaddicted families (Tunca et al. 2024; Happ et al. 2023), while interdependence theory emphasised how reduced marital power contributes to co‐dependent patterns (Atintas and Tutarel‐Kislak 2019).

Liverano et al. (2023) conceptualised co‐dependency as a form of love addiction, characterised by a parent–child dynamic. While studies like Lampis et al. (2017) suggest similar dynamics, they did not frame these within love addiction models. Other scholars challenged the pathologisation of co‐dependency. For example, stress‐coping models proposed co‐dependent behaviours as relational adaptations (Calderwood and Rajesparam 2014), with others introduced caregiver burnout as an alternative framing (Shishkova and Bocharov 2022). Consistent with these interpretations, Ehsan and Suneel (2020) found co‐dependency prevalent in parents of disabled children.

#### 3.4.4 Addiction and Pathology Perspectives

This conceptualisation situates co‐dependency as a disorder, commonly framed through addiction or pathology models. Vederhus et al. (2019) described co‐dependency as a maladaptive response to addiction‐related stress, characterised by emotional suppression. Rozhnova et al. (2020) framed co‐dependency as a nonchemical addiction and found higher rates of alcoholism among co‐dependents' relatives, highlighting potential hereditary factors. Zielinski et al. (2019) identified a potential neurological basis for co‐dependency, observing reduced brain activation in the dorsomedial prefrontal cortex.

Pathological frameworks beyond addiction also persist. Knapek et al. (2017, 2021) found co‐dependency to be distinct from personality disorders but often retained a substance‐related framing by describing individuals as SUD partners. Liverano et al. (2023) expanded this perspective by conceptualising co‐dependency as a relational addiction, challenging narrow substance‐use interpretations.

#### 3.4.5 Developmental Perspectives

This perspective conceptualises co‐dependency through developmental frameworks, locating over functioning and enmeshment in early attachment disruptions. Weiss (2019) introduced the concept of *Prodependence*, reframing co‐dependency as a normative expression of attachment needs. Coffman and Swank (2021) expanded on this, linking insecure attachment in families affected by SUD to relational overfunctioning. Liverano et al. (2023) further conceptualised co‐dependency as rooted in disorganised and anxious attachment. Though limited, empirical studies reinforced these associations, showing a stronger relationship between co‐dependency and attachment anxiety (Chang 2018; Tunca et al. 2024), alongside a weaker association with attachment avoidance. Notably, Tunca et al. (2024) also found that secure attachment predicted co‐dependency in nonclinical populations.

Trauma models also situate co‐dependency in early adversity. Qualitative studies (Bacon et al. 2020; Klimczak and Klejna 2018) highlighted childhood neglect and loss of control. Quantitative findings (EvgIn and Sümen 2022; Kaya et al. 2024) confirmed associations between emotional abuse, neglect and co‐dependency. Kaya et al. also found resilience buffered the impact of abuse but not neglect.

#### 3.4.6 Psychoanalytic Perspectives

This perspective frames co‐dependency as a defence rooted in early relational experiences. Bacon et al. (2020) applied Winnicott's false self‐theory, framing co‐dependency as a defensive adaptation to childhood invalidation, where individuals self‐suppress to meet external demands. Liverano et al. (2023) extended this through Freud's repetition compulsion, suggesting co‐dependents unconsciously recreate early relational dynamics to resolve unmet needs.

Recent studies drew on Kohut's self‐psychology to inform their conceptualisations. Sobol‐Goldberg et al. (2024) used mirroring theory to argue that disrupted early relationships impair self‐cohesion, fostering dependency. Kaplan (2023) applied self‐perception theory to describe how caregiving roles reinforce ‘saviour’ identities. Finally, studies by Tunca et al. (2024) and Aristizábal (2020) foregrounded immature defence mechanisms as central to co‐dependency, helping individuals manage relational anxiety.

#### 3.4.7 Cognitive‐Personality Perspectives

This perspective conceptualises co‐dependency through personality and cognitive psychology. Studies examined traits, schemas and emotional processes associated with co‐dependency. Hawkins and Hawkins (2014) positioned co‐dependency and contradependence on a continuum of maladaptive interpersonal styles, underscoring the tension between autonomy and connection. Kolenova et al. (2023) framed co‐dependency as a stable personality attitude shaped by diverse cognitive and emotional patterns while Klimczak and Klejna (2018) identified a common Big Five profile, suggesting converging traits across co‐dependent individuals.

Schema theory was explicitly applied in recent studies. Bacon and Conway (2023) and Knapek et al. (2021) identified maladaptive schemas involving impaired autonomy, self‐sacrifice and self‐subjugation. Karasar (2020) linked co‐dependency to perfectionism and the pursuit of social approval, echoing schemas tied to shame and defectiveness. Emotional dysregulation was also prominent. Rozhnova et al. (2020) described emotional suppression, overload and ‘auto‐aggressive’ behaviours while Bespalov et al. (2024) suggested that emotional intelligence deficits may underpin such difficulties.

### 3.5 The Impact of Co‐Dependency on Well‐Being

To address our second question (What mental health outcomes are associated with co‐dependency?), this section synthesises empirical literature on the mental health impact of co‐dependency. While many studies discussed potential consequences of co‐dependency, only the 11 included studies included measured outcomes (see Supplementary File 5, Table E2 for study‐to‐theme mapping).

Several studies reported outcomes related to social functioning and self‐perception. While not always classified as clinical disorders, these outcomes are increasingly recognised as relationally embedded psychological states that influence well‐being (Feeney and Collins 2015). To capture this broader understanding, three subthemes were identified (Table 5), illustrating the multifaceted impact of co‐dependency.

#### 3.5.1 Self‐Concept and Identity

Many studies identified difficulties with identity and self‐concept as both causes and consequences of co‐dependency. Chang (2018) framed low self‐esteem as a defining feature of co‐dependency. Others (e.g., EvgIn and Sümen 2022) highlighted how co‐dependency erodes self‐worth through identity loss. Similarly, Kaplan (2023) found that housewives often internalised caregiving roles at the expense of their own needs, reinforcing negative self‐perception. Sobol‐Goldberg et al. (2024) highlighted self‐stigma, leading to identity confusion.

#### 3.5.2 Relational and Social Functioning

Theme 1 focuses on the impact of co‐dependency on relational and social functioning. Chang (2018) reported associations between co‐dependency and impaired social adjustment. Vederhus et al. (2019) found that co‐dependency contributed to long‐term family dysfunction, while Atintas and Tutarel‐Kislak (2019) linked it to reduced marital power and satisfaction. Aristizábal (2020) reported that participants described co‐dependency within violent relationships, alongside feelings of isolation. Zielinski et al. (2019) added a neuropsychological perspective, identifying executive dysfunction and heightened relational stress, which might impair co‐dependents' ability to regulate social interactions.

#### 3.5.3 Emotional and Psychological Wellbeing

Beyond identity and relationships, co‐dependency is associated with emotional and psychological distress. Chang (2018) found that psychological adjustment problems are central to co‐dependency while Rozhnova et al. (2020) linked co‐dependency with somatoform disorders and emotional vulnerability.

Depression, anxiety, emotional dysregulation and trauma symptoms were reported (Aristizábal 2020; Atintas and Tutarel‐Kislak 2019; Ehsan and Suneel 2020; EvgIn and Sümen 2022; Kaplan 2023), with guilt and shame highlighted by Nordgren et al. (2020) and Sobol‐Goldberg et al. (2024). Zielinski et al. (2019) further identified biological factors contributing to emotional instability.

Unsurprisingly, overall well‐being is impaired. Vederhus et al. (2019) and Happ et al. (2023) reported reduced life satisfaction and quality of life. These difficulties were sometimes linked to maladaptive coping such as substance use (EvgIn and Sümen 2022; Rozhnova et al. 2020) and crime involvement (Aristizábal 2020).

### 3.6 Confidence in Review Findings

Four findings were rated as high confidence, while five were rated as moderate or moderate–high. Moderate ratings reflected minor conceptual inconsistencies or limited data depth in specific domains (e.g., developmental and cognitive‐personality perspectives). Overall, the evidence base was robust, with most findings supported by multiple studies across methods and contexts. A full table is provided in the Supplementary File 6 (Table F1).

## 4 Discussion

### 4.1 Summary of Findings

This review highlights the ongoing conceptual fragmentation of co‐dependency, aligning with previous critiques of its ambiguity (e.g., Hands and Dear 1994). However, rather than dismissing the construct's utility, our review also evidences a pattern of recurrent mental health challenges noted in the literature (Martsolf et al. 2000), underscoring its clinical relevance.

While diverse frameworks offer insight, these perspectives remain fragmented. For example, relational perspectives emphasise enmeshment and low self‐differentiation, while psychological approaches foreground schemas and emotional dysregulation. Trauma‐informed studies highlight early adversity (e.g., EvgIn and Sümen 2022), yet debates persist over whether this pathologises caregiving (Weiss 2019) or restore agency by contextualising distress (Kaya et al. 2024).

Definitional inconsistency also persists. For example, Kolenova et al. (2023) defined co‐dependency as a multidimensional personality attitude, whereas Klimczak and Klejna (2018) identified a common Big Five profile. Similarly, Liverano et al. (2023) conceptualised co‐dependency as a form of love addiction, while other studies suggested comparable dynamics (e.g., Kaya et al. 2024; Lampis et al. 2017) without explicitly engaging with love addiction frameworks. Recent empirical work has also linked co‐dependency with impulsivity and behavioural addiction processes (Diotaiuti, Mancone, et al. 2022), lending further support to addiction‐oriented interpretations. This raises questions about whether co‐dependency constitutes a distinct construct, a personality disorder–adjacent phenomenon (Knapek et al. 2017, 2021), or part of a continuum with related constructs such as love addiction. Rather than resolving this debate, the present review highlights the need for integrative frameworks that can accommodate complexity.

Across the literature, mental health outcomes clustered around identity disruption, psychological distress and dysfunctional relationships. Importantly, these outcomes were sometimes described both as causes and consequences of co‐dependency (e.g., Chang 2018). Although impaired self‐esteem has traditionally been identified as a core characteristic of co‐dependency (Cermak 1986), several studies in this review (e.g., Kaplan 2023) positioned it as an outcome. However, as most of these studies were correlational, causality cannot be inferred.

Within the broader domain of psychological distress, emotional dysregulation was reported across studies (e.g., Rozhnova et al. 2020; Zielinski et al. 2019), although often described in broad terms rather than as specific regulatory processes. This pattern is further elaborated by Diotaiuti, Girelli, et al. (2022), who linked addiction with impulsivity and ruminative processes, highlighting how deregulatory processes may contribute to and potentially sustain dependency‐related distress.

Despite the consistent association to distress, many studies framed co‐dependent behaviours as adaptive. Across trauma‐ and attachment‐informed studies, behaviours such as self‐suppression, overfunctioning and relational hypervigilance emerged as strategies that were self‐protective within adverse caregiving environments (e.g., Bacon et al. 2020; Coffman and Swank 2021; Weiss 2019). Relational perspectives similarly interpreted co‐dependent behaviours as stabilising responses within dysfunctional family systems (e.g., Lampis et al. 2017; Tunca et al. 2024) while sociocultural studies highlighted how self‐sacrifice and emotional labour may be socially rewarded and therefore adaptive within patriarchal or collectivist contexts (e.g., Kaplan 2023; Atintas and Tutarel‐Kislak 2019). Even in addiction contexts, emotional suppression and hypervigilance were often described as coping responses rather than pathology (Vederhus et al. 2019).

### 4.2 Towards an Integrative Conceptualisation of Co‐Dependency

To address the fragmented literature, two integrative frameworks are proposed.

**FIGURE 2:**
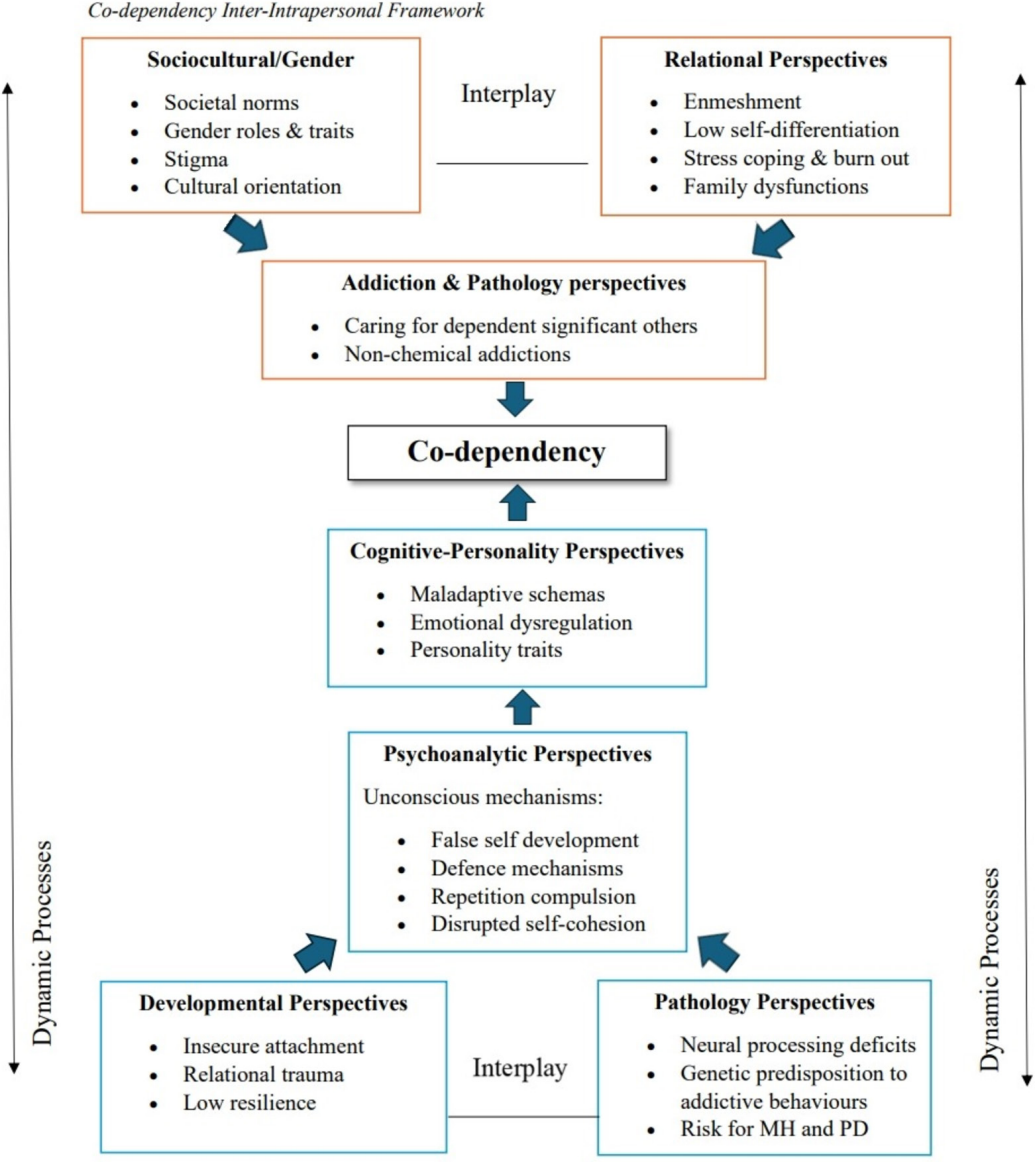
The inter–intrapersonal framework of co‐dependency. Red text boxes represent interpersonal factors, blue text boxes represent intrapersonal factors, and dashed boxes indicate areas of conceptual debate spanning multiple perspectives. One‐directional arrows illustrate hypothesised influences on co‐dependent patterns, while bi‐directional arrows highlight dynamic and reciprocal feedback processes between interpersonal and intrapersonal mechanisms.

Figure 2 integrates diverse perspectives into a tentative framework outlining how co‐dependency may develop and be maintained. At its base, developmental vulnerabilities such as insecure attachment and trauma create internal templates for relating (Bacon et al. 2020; Bowlby 1969; Coffman and Swank 2021). These may interact with biological predisposition, including susceptibility to mental health conditions, addiction and neural processing differences (Rozhnova et al. 2020; Zielinski et al. 2019).

Psychodynamic processes, including defences and repetition compulsion (Freud 1938), might arise from and reinforce these vulnerabilities, contributing to the re‐enactment of familiar relational patterns (Tunca et al. 2024; Liverano et al. 2023). Overtime, such processes may contribute to the formation of maladaptive schemas (Young et al. 2003) and emotional dysregulation (Bacon and Conway 2023). Personality traits (Costa and McCrae 1992), while relatively stable, may interact with schemas and relational dynamics to amplify vulnerability or resilience (Bacon and Conway 2023).

These intrapersonal dynamics are likely activated and sustained within interpersonal experiences. Research highlights family enmeshment, low self‐differentiation (Bowen 1978; Chang, 2019; Lampis et al. 2017; Minuchin 1974) and caregiving stress (Ehsan and Suneel 2020), while sociocultural norms can reinforce self‐sacrifice and overfunctioning (e.g., Kaplan 2023). Addiction‐based models position co‐dependency as a relational or behavioural addiction, emerging within relational contexts marked by stress and unpredictability (e.g., Knapek et al. 2017, 2021). The framework therefore tentatively emphasises reciprocal feedback loops rather than linear causation.

**FIGURE 3:**
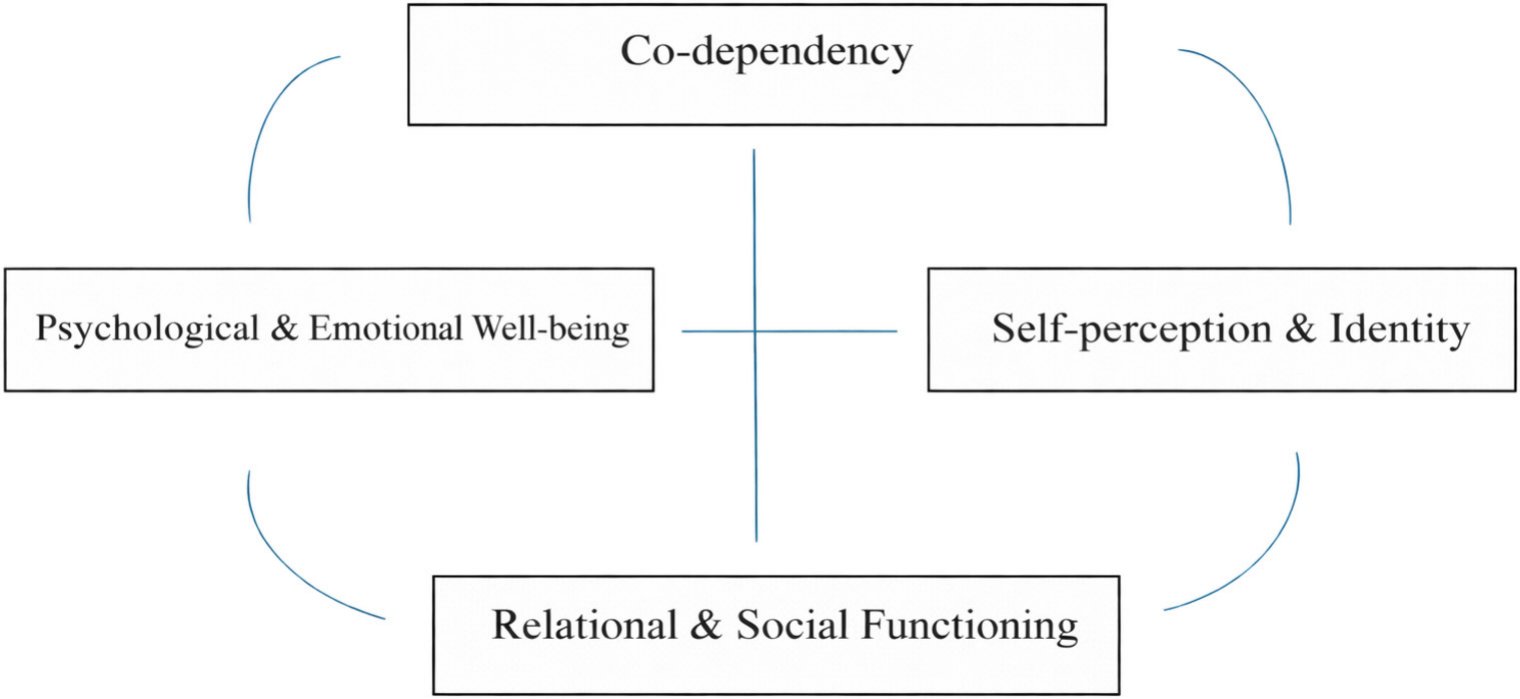
Interconnected mental health outcomes of co‐dependency. This framework illustrates how co‐dependency is associated with psychological well‐being, self‐perception and relational functioning. These domains are likely interconnected, such that challenges in one area may reinforce difficulties in others, contributing to the maintenance of co‐dependent patterns.

Figure 3 complements this perspective by organising co‐dependency mental health outcomes. Difficulties in self‐concept, emotional and psychological distress and impaired relational functioning appear to interact in self‐perpetuating ways. While low self‐esteem and relational difficulties have often been described as inherent features of co‐dependency (Chang 2018), this synthesis suggests they may also emerge as outcomes of sustained relational stress. EvgIn and Sümen (2022) showed that neglect within co‐dependent dynamics eroded self‐worth and increased vulnerability to maladaptive coping, while Kaplan (2023) highlighted how internalised caregiving roles reinforced negative self‐perception. Aristizábal (2020) further described how co‐dependency may sustain violent relationships, contributing to shame and isolation.

In turn, these outcomes can reinforce dependency itself: Reduced life satisfaction (Vederhus et al. 2019; Happ et al. 2023), emotional dysregulation (Zielinski et al. 2019) and shame (Sobol‐Goldberg et al. 2024; Nordgren et al. 2020) may increase reliance on external validation, reinforcing co‐dependent behaviours in a self‐perpetuating cycle.

Together, these findings support conceptualising co‐dependency as a dynamic relational process rather than a static trait or disorder.

### 4.3 Strengths and Limitations

This review's key strength lies in its integrative approach and methodological rigour, supported by investigator triangulation. The development of two original frameworks (Figures 2 and 3) makes a novel theoretical contribution by structuring a fragmented literature base.

Challenges included managing a large and diverse evidence base. Limiting the timeframe to the past 10 years ensured relevance but excluded earlier foundational work. Screening criteria were refined to improve rigour (Higgins and Thomas 2020), though early‐stage exclusions may benefit from clearer guidance in future reviews. It should be noted that no attempts were made to contact study authors for additional information. While this was not considered necessary given the level of detail reported in the included studies, it may have marginally limited the completeness of extracted data.

Inconsistent terminology complicated the full‐text screening process. Although adjacent terms were included in the search strategy, studies were only retained if co‐dependency was explicitly referenced. While this ensured conceptual focus and consistency, it may have narrowed the scope of insights. Future research could examine related constructs in parallel to improve clarity and capture overlapping phenomena.

The risk of bias was limited due to quality appraisal procedures. However, as this was conducted by a single reviewer, some risk of subjectivity remains. While resource constraints made double appraisal unfeasible, transparency was maintained by using structured tools and extraction templates. Additionally, the empirical studies differ greatly in focus and sample, which may affect the generalisability of findings. Geographic concentration of the studies further limits generalisability. While some studies explored culture (e.g., Lampis et al. 2017), this remained underexamined. Future research could focus on the role of cultural norms in shaping co‐dependent behaviours.

Although attachment theory has provided valuable insights, empirical research in this area remains limited and inconsistent. Research on the mental health outcomes is more extensive but lacks systematic exploration, particularly concerning potential adaptive or growth‐oriented aspects. Future studies should investigate attachment processes in greater depth and consider the possibility of posttraumatic growth, supporting a more balanced and clinically meaningful understanding.

A further limitation is that most of the quantitative research was correlational in design, meaning that causality cannot be inferred. This limits the ability to distinguish antecedents, correlates and consequences of co‐dependency and reflects the limited availability of longitudinal or experimental studies able to examine developmental pathways or change over time. Similarly, much of the existing research currently relies heavily on self‐report measures, which may be influenced by social desirability and norms around caregiving and self‐sacrifice. This is particularly relevant for a construct such as co‐dependency, where behaviours may be socially reinforced or normalised within specific contexts. These issues are especially pertinent to the evidence on mental health outcomes, where only 11 studies reported measured effects, further restricting the strength of conclusions regarding directionality and impact.

Finally, qualitative and conceptual contributions remain underreported and underrepresented in the literature. Despite their relevance for theory development and meaning‐making, such studies were relatively few and often difficult to locate due to indexing limitations. Broader database indexing and inclusive search strategies may be needed to ensure these perspectives are captured in future syntheses.

### 4.4 Clinical Implications

These findings underscore the importance of understanding psychological distress through a relational and formulation‐based lens, emphasising the interaction between internal vulnerabilities and recurring interpersonal patterns. This supports the need for a strengths‐based, integrative approach to co‐dependency that reframes behaviours often pathologised as potentially adaptive coping strategies developed in the context of relational trauma.

Given evidence that resilience alone may not protect against the effects of early neglect (Kaya et al. 2024), interventions should address underlying shame, unmet emotional needs and attachment disruptions. Although this review did not directly evaluate interventions, approaches such as attachment‐based therapy (Wallin 2007), cognitive analytic therapy (Ryle and Kerr 2020) and schema therapy (Young et al. 2003) may be relevant, given their focus on relational patterns, reciprocal roles and entrenched schemas. Furthermore, early access to family therapy may be beneficial in contexts of dysfunction, while safeguarding professionals should be encouraged to recognise co‐dependent dynamics and offer appropriate signposting. Trauma‐focused or psychodynamic approaches may also support clients in processing unresolved relational trauma and repetition compulsion. Future research should evaluate their applicability for co‐dependency more systematically.

### 4.5 Policy Implications

This review highlights the ongoing lack of conceptual clarity surrounding co‐dependency yet points to consistent evidence of its negative psychological outcomes. Rather than framing co‐dependency as a diagnostic category, it should be recognised as a maladaptive relational coping pattern that often emerges in the context of early adversity.

A key first step for recognition at the policy and service level is the development of validated screening measures, as current tools remain limited to research contexts. Reliable, clinically validated measures would enable practitioners to identify co‐dependent patterns within routine assessments and formulations.

Policymakers could support commissioning research into its prevalence, developmental pathways and cultural relevance. Public health campaigns and school‐based initiatives could support prevention by teaching emotional and relational literacy, for example, boundary‐setting and reciprocity skills. Digital and interactive health literacy approaches have shown promise in adolescent populations (Mancone et al. 2024), suggesting potential value in adapting such models to promote relational literacy and prevent maladaptive dependency patterns. Parenting programmes could also be adapted to address relational overfunctioning and promote emotional validation.

Funding should also support the development of targeted interventions. Given the reported value of peer‐led recovery groups such as CoDA, clearer referral pathways between statutory services and community‐based support are needed.

### 4.6 Research Directions

The field should move beyond narrow, addiction‐based conceptualisations of co‐dependency and address the growing relational and developmental perspectives. The frameworks proposed in this review (Figures 2 and 3) provide a foundation for further empirical development.

A necessary first step is the operationalisation and validation of the preliminary frameworks. An interpersonal–intrapersonal framework (Figure 2) could be operationalised through validated measures of attachment, early trauma, schemas and family functioning, examined in multivariate or longitudinal models. If tested, this framework (Figure 2) could inform the creation of updated assessment tools that capture both intrapersonal and interpersonal dimensions.

Similarly, the mental health outcomes framework (Figure 3) could be validated by exploring mediational pathways linking self‐concept, distress and relational functioning. Research could also assess the role of attachment strategies and cultural values, helping to clarify contextual influences.

## 5 Conclusion

This review synthesised recent evidence on co‐dependency and introduced two integrative frameworks that illuminate its diverse conceptualisations and associated mental health outcomes. Findings underscore co‐dependency as a complex, multidimensional construct with significant implications for psychological well‐being.

This review critiques the field's tendency to pathologise co‐dependency and advocates for integrative, strengths‐based perspectives. Key limitations identified include ongoing conceptual ambiguity, limited cultural contextualisation and inconsistencies in attachment‐related findings. Future research should clarify distinctions between co‐dependency and overlapping constructs, investigate cultural influences and explore developmental trajectories across the lifespan.

Clinically, the findings point to the potential value of trauma‐ and attachment‐informed interventions that are culturally sensitive. The preliminary frameworks proposed here may, with further validation, offer a foundation for informing practice and policy.

## Supporting information


**Supplementary File 1:** provides the full search strategies, including database‐specific search strings and applied filters.
**Supplementary File 2:** presents the list of studies excluded at full‐text screening with reasons for exclusion.
**Supplementary File 3:** contains detailed data extraction tables for all included studies.
**Supplementary File 4:** summarises the quality appraisal outcomes using MMAT and JBI tools.
**Supplementary File 5:** presents the narrative grouping and study‐to‐theme mappings.
**Supplementary File 6:** provides the certainty/confidence assessment for key review findings.

## Data Availability

Data sharing is not applicable to this article as no new data were created or analysed in this study.
