# Supplementary material for: Co‐Dependency Revisited: An Integrative Review of Conceptualisations and Mental Health Outcomes
Source: Clin Psychol Psychother. 2026 Apr 10;33(2):e70265. doi: 10.1002/cpp.70265 (PMC13067074; doi:10.1002/cpp.70265)
Supplement: Supplementary file 1 — provides the full search strategies, including database‐specific search strings and applied filters. Supplementary File 2: presents the list of studies excluded at full‐text screening with reasons for exclusion. Supplementary File 3: contains detailed data extraction tables for all included studies. Supplementary File 4: summarises the quality appraisal outcomes using MMAT and JBI tools. Supplementary File 5: presents the narrative grouping and study‐to‐theme mappings. Supplementary File 6: provides the certainty/confidence assessment for key review findings. [file CPP-33-e70265-s001.docx]

**SUPPLEMENTARY MATERIALf**

**Supplementary File 1. Search Strategy**……………………………………………………………**2-4**

**Supplementary File 2: Studies Excluded at Full-text Screening**……………………………….**.5-14**

**Supplementary File 3. Extraction Tables**……………………….………………………………**15-30**

**Supplementary File 4. Quality Appraisal**……………………….………………………………**31-33**

**Supplementary File 5. Narrative Grouping**……………………….……………………………**34-37**

**Supplementary File 6. Certainty Assessment**……………………….……………………………...**38**

**Supplementary File 1. Search Strategy**

The same core search string was applied across all databases with minor syntax adjustments based on database requirements.

**Search string used:** ("codependen*" OR "co-dependen*" OR "love addiction" OR "enmeshment" OR "relationship addiction" OR "affective dependenc*" OR "relational dependenc*") AND ("conceptualisation" OR "definition" OR "understanding" OR "theoretical model" OR "conceptual framework" OR "psychological theory" OR "model of co-dependen*" OR "theoretical construct") AND ("mental health outcomes" OR anxiety OR depression OR stress OR "well-being" OR "psychological impact")

During the database searches, it became evident that a single search combining both conceptualisation and mental health outcome terms yielded low precision or overly broad results. Therefore, in databases such as PubMed, PsycINFO, and ProQuest, the search was strategically split into two parallel queries:

- One focusing on conceptualisations of co-dependency (e.g., theoretical models, definitions, frameworks).
- One focusing on mental health outcomes (e.g., anxiety, depression, well-being).

This approach allowed for more focused retrieval of relevant studies for each review question and reduced the inclusion of irrelevant records. Final search strings are reported in Supplementary Table A1.

**Filters applied in all databases:**

- Language: English
- Sample: Adults
- Date range: No date restrictions were applied during the initial database search. During the screening stage, a decision was made to include only studies published between 2013 and 2024, in order to focus on recent conceptual developments and avoid duplication of findings.

**Table A1**

Final Search Strings

| **Database** | **String** | **Filters** |
| --- | --- | --- |
| EBSCO | Search 1: ("co-dependency" OR "codependency") AND ("concept*" OR "definition" OR "theor*" OR "understanding" OR "trends")  Search 2: ("co-dependency" OR "codependency" OR "love addiction" OR "relationship addiction" OR "relational dependency") AND ("mental health outcomes" OR "anxiety" OR "depression" OR "stress" OR "well-being" OR "psychological impact") | None beyond standard filters already stated |
| PubMED | ("co-dependency" OR "codependency" OR "love addiction" OR "relationship addiction") AND ("conceptualisation" OR "definition" OR "understanding" OR "theoretical model" OR "conceptual framework" OR "psychological theory" OR "model of co-dependen*" OR "theoretical construct") AND ("mental health outcomes" OR "anxiety" OR "depression" OR "stress" OR "well-being" OR "psychological impact") NOT ("affective disorder" OR "bipolar" OR "COVID-19" OR "pregnancy" OR "bone*" OR "signal*" OR "Alzheimer*" OR "drug dependen*") | Humans |
| PsychINFO | Search 1: ("co-dependen*" OR "codependen*" OR "love addiction" OR "relationship addiction" OR "affective dependenc*" OR "relational dependenc*") AND ("conceptualization" OR "definition" OR "understanding" OR "interpretation" OR "theoretical model" OR "theoretical framework" OR "conceptual framework" OR "theoretical perspective" OR "psychological theory" OR "model of co-dependency" OR "theoretical construct")  Search 2: ("co-dependen*" OR "codependen*" OR "love addiction" OR "relationship addiction" OR "affective dependenc**" OR "relational dependenc*") AND ("conceptualization" OR "definition" OR "understanding" OR "interpretation" OR "theoretical model" OR "theoretical framework" OR "conceptual framework" OR "theoretical perspective" OR "psychological theory" OR "model of co-dependency" OR "theoretical construct")  Search 3: ("co-dependency" OR "codependency" OR "love addiction" OR "relationship addiction" OR "relational dependency")  AND  ("mental health outcomes" OR "anxiety" OR "depression" OR "stress" OR "well-being" OR "psychological impact") | None beyond standard filters already stated |
| PsychArticles | ("co-dependency" OR "codependency OR ''love addiction'' OR ''relationship addiction'') | None beyond standard filters already stated |
| ProQuest Central | Search 1: ("co-dependen*" OR "codependen*" OR "love addiction" OR "relationship addiction" OR "affective dependenc*" OR "relational dependenc*")  AND  ("conceptual*" OR "definition" OR "understanding" OR "theoretical model" OR "theoretical framework" OR "psychological theory" OR "theoretical construct")  NOT  (metabol* OR terrorism OR withdrawal OR law OR biological OR dementia OR energy OR sedentar* OR pain OR business OR colonialism OR blood OR musculoskeletal OR genetic)  Search 2: ("co-dependency" OR "codependency" OR "love addiction" OR "relationship addiction" OR "relational dependency")  AND  ("mental health outcomes" OR "anxiety" OR "depression" OR "stress" OR "well-being" OR "psychological impact")  NOT  (metabol* OR terrorism OR withdrawal OR law OR biological OR dementia OR energy OR sedentar* OR pain OR business OR colonialism OR blood OR musculoskeletal OR "structure sensitivity" OR genetic) | None beyond standard filters already stated |
| ProQuest Dissertations & Theses Global | ("co-dependency" OR "codependency" OR "love addiction" OR "relationship addiction" OR "enmeshment" OR "relational dependency")  AND  ("conceptualization" OR "definition" OR "understanding" OR "interpretation" OR "theoretical model" OR "theoretical framework" OR "conceptual framework" OR "theoretical perspective" OR "psychological theory" OR "model of co-dependency" OR "theoretical construct")  AND  ("mental health outcomes" OR "anxiety" OR "depression" OR "stress" OR "well-being" OR "psychological impact")  NOT  ("entrepreneur*" OR "veterans" OR "chronic" OR "technology") | None beyond standard filters already stated |
| Scopus | Search 1: ("co-dependency" OR "codependency") AND ("concept*" OR "definition" OR "theor*" OR "understanding" OR "trends")  Search 2: ("co-dependency" OR "codependency") AND ("mental health outcomes" OR "wellbeing") | None beyond standard filters already stated |

**Supplementary File 2: Studies Excluded at Full-text Screening**

**Table B1**

| **Study** | **Reason for Exclusion** |
| --- | --- |
| Abadi et al., 2015 | Main focus on intervention |
| Aguila, 2019 | Grey literature with insufficient conceptual depth |
| Ahmad-Abadi et al., 2017 | Main focus on intervention |
| Aimaganbetova et al, 2020 | Insufficient conceptual depth |
| Alpsoy, 2023 | Grey literature; initially eligible but excluded in favour of peer-reviewed studies due to high volume of included articles. |
| Artemtseva & Malkina, 2022 | Not available in English |
| Askian, 2021 | Wrong study design |
| Atlam et al., 2023 | Co-dependency not the primary focus |
| Bacon, 2016 | Duplicate findings |
| Bacon et al., 2021 | Main focus on intervention |
| Bala & Devaiah, 2019 | Insufficient conceptual depth |
| Balliet et al., 2017 | Related term, no mention of co-dependency |
| Bliss, 2022 | Grey literature with Insufficient conceptual depth |
| Bortolon et al., 2016 | Insufficient conceptual depth |
| Carias & Granato, 2023 | Main focus on SUD |
| Caridade et al., 2020 | Co-dependency not the primary focus |
| Chang & Bhat, 2023 | Main focus on intervention |
| Çimşir & Akdoğan, 2021 | Co-dependency not the primary focus |
| Costa et al., 2021 | Related term, no mention of co-dependency |
| Costa & Oliveira-Monteiro, 2020 | Wrong population (Adolescents) |
| Dineen & Dinc, 2024 | Related term, no mention of co-dependency |
| Diotaiuti et al., 2022 | Co-dependency not the primary focus |
| Danilov & Walsh, 2023 | Co-dependency not the primary focus |
| Earp et al., 2017 | Related term, no mention of co-dependency |
| Ermakov et al., 2019 | Insufficient conceptual depth |
| Escuza Pasco & Chunga Diaz, 2022 | Main focus on intervention |
| Esmaeilpour, 2024 | Co-dependency not the primary focus |
| Fard et al., 2019 | Main focus on SUD |
| Fernandez et al., 2021 | Related term, no mention of co-dependency |
| Giacobbe et al., 2024 | Related term, no mention of co-dependency |
| Gomberg, 2019 | Wrong format |
| Gori et al., 2023 | Related term, no mention of co-dependency |
| Gori et al., 2024 | Related term, no mention of co-dependency |
| Graham, 2022 | Related term, no mention of co-dependency |
| Grier, 2022 | Grey literature with Insufficient conceptual depth |
| Grigoviera, 2020 | Not available in English |
| Grunko et al., 2019 | Main focus on SUD |
| Hall, 2013 | Related term, no mention of co-dependency |
| Hentsch-Cowles & Brock, 2013 | Co-dependency not the primary focus |
| Hill, 2023 | Related term, no mention of co-dependency |
| Hodge, 2015 | Main focus on SUD |
| Kaur, 2016 | Overlapping findings |
| Kemer et al., 2016 | Related term, no mention of co-dependency |
| Kim et al., 2021 | Co-dependency not the primary focus |
| Knapek & Szabó, 2014 | Not available in English |
| Kolenova et al., 2021 | Insufficient conceptual depth |
| Kolenova et al., 2023 | Duplicate findings |
| Kornaszewska-Polak, 2019 | Insufficient conceptual depth |
| Lee & Kim, 2016 | Main focus on intervention |
| Lemos, & Román-Calderón, 2019 | Related term, no mention of co-dependency |
| Lymankina, 2023 | Insufficient conceptual depth |
| Maglia et al., 2023 | Related term, no mention of co-dependency |
| Maina, 2021 | Main focus on SUD |
| Margasiński, 2022 | Co-dependency not the primary focus |
| Mashuri & Patriasih, 2023 | Related term, no mention of co-dependency |
| Momeñe et al., 2022 | Related term, no mention of co-dependency |
| Navarro, 2023 | Main focus on SUD |
| Odokonyero et al., 2022 | Wrong study design |
| Orsolini et al., 2022 | Related term, no mention of co-dependency |
| Özal et al., 2023 | Related term, no mention of co-dependency |
| Pant et al., 2022 | Main focus on SUD |
| Pekyiğit et al., 2024 | Insufficient conceptual depth |
| Petrucelli et al., 2014 | Co-dependency not the primary focus |
| Pugliese et al., 2023 | Related term, no mention of co-dependency |
| Redcay & McMahon, 2021 | Related term, no mention of co-dependency |
| Rhodes et al., 2017 | Insufficient conceptual depth |
| Rice, 2017 | Wrong format |
| Roberts, 2016 | Co-dependency not the primary focus |
| Roberts, 2021 | Co-dependency not the primary focus |
| Rodríguez Blanco, 2013 | Wrong study design |
| Ruiz-García et al., 2021 | Co-dependency not the primary focus |
| Sanches & John, 2019 | Related term, no mention of co-dependency |
| Salani et al., 2022 | Related term, no mention of co-dependency |
| Sarkar et al., 2015 | Insufficient conceptual depth |
| Sirvent-Ruiz et al., 2022 | Insufficient conceptual depth |
| Spivakovskaya & Lutsenko, 2021 | Main focus on SUD |
| Tekin & Ançel, 2023 | Overlapping findings |
| Turan et al., 2021 | Insufficient conceptual depth |
| Ulusoy & Güçray 2019 | Main focus on intervention |
| Vasilyeva, 2023 | Main focus on intervention |
| Walters & Simons, 2023 | Related term, no mention of co-dependency |
| Weegmann & Head, 2016 | Co-dependency not the primary focus |
| Weiss, 2018 | Wrong format |
| Weiss, 2022 | Duplicate findings |
| Woolfolk, 2017 | Wrong format |
| Wnuk, 2015 | Insufficient conceptual depth |
| Wnuk, 2023 | Insufficient conceptual depth |
| Wright et al., 2021 | Related term, no mention of co-dependency |
| Yang & Hong, 2015 | Not available in English |
| Young & Timko, 2015 | Co-dependency not the primary focus |
| Yoshii 2023 | Not available in English |
| Zaidi, 2015 | Insufficient conceptual depth |
| Zerbetto et al., 2024 | Insufficient conceptual depth |

**References**

Abadi, F. K. A., Vand, M. M., & Aghaee, H. (2015). Models and interventions of codependency treatment, systematic review. *Journal UMP Social Sciences and Technology Management*, *3*(2).

Aguila, B. R. (2019). Codependency traits and the mere presence of a cell phone.

Ahmad-Abadi, F. K., Maarefvand, M., Aghaei, H., Hosseinzadeh, S., Abbasi, M., & Khubchandani, J. (2017). Effectiveness of Satir-informed family-therapy on the codependency of drug dependents’ family members in Iran: a randomized controlled trial. *Journal of evidence-informed social work*, *14*(4), 301-310.

Alpsoy, P. (2023). The mediating role of selfobject needs and psychological needs on the relationship between attachment styles and codependency (Master's thesis, İzmir Ekonomi Üniversitesi).

Aimaganbetova, O., Syrgakbaeva, A., Zhantikeyev, S., Lashkova, Y., Nurbekova, Z., & Zhanazarova, Z. (2020). Study of family system characteristics in co-dependent women. *Archives of Psychiatry & Psychotherapy*, *22*(3).

Artemtseva, N. G., & Malkina, M. A. (2022). Cognitive mistakes of codependents as a way to protect against uncertainty. *Vestnik Of Samara State Technical University Psychological And Pedagogical Sciences*, *19*(1), 153-166.

Atlam, D., Akyel-Göven, B., & Yüncü, Z. (2023). Addiction in romantic relationships scale development: validity and reliability study. Addicta: The Turkish Journal on Addictions, 10(1), 52-58.

Askian, P., Krauss, S. E., Baba, M., Kadir, R. A., & Masoumian Sharghi, H. (2021). Recovering from co-dependence: A study of iranian wives of persons with substance use disorder. *Current Psychology*, *40*, 1234-1248.

Bacon, I. (2016). ‘Like a seesaw, I feel out of control!’An exploration of the lived experience of codependency through IPA.

Bacon, I., McKay, E., Reynolds, F., & McIntyre, A. (2021). An examination of the lived experience of attending twelve-step groups for co-dependency. *International Journal of Mental Health and Addiction*, *19*, 1646-1661.

Bala, A., & Devaiah, D. (2019). Mediating Role of Co-dependency on Age and Victimisation Experiences among Indian Employees. Ushus Journal of Business Management, 18(2), 1-21.

Balliet, D., Tybur, J. M., & Van Lange, P. A. (2017). Functional interdependence theory: An evolutionary account of social situations. *Personality and Social Psychology Review*, *21*(4), 361-388.

Bliss, B. (2022). Disentangling Codependency from Empathy: A Steinian Trinitarian Account of the Healing of Personhood. *Lumen et Vita*, *12*(2), 14-27.

Bortolon, C. B., Signor, L., Moreira, T. D. C., Figueiró, L. R., Benchaya, M. C., Machado, C. A., ... & Barros, H. M. T. (2016). Family functioning and health issues associated with codependency in families of drug users. *Ciência & saude coletiva*, *21*(1), 101-107.

Carias, A. R., & Granato, T. M. M. (2023). Theoretical-methodological rationales in caring for family members of people with alcohol use disorders. *Estudos de Psicologia (Campinas)*, *40*, e200180.

Caridade, S., Pinheiro, I., & Dinis, M. A. P. (2020). Stay or leave abusive dating relationships: Portuguese victims’ reasons and barriers. *Social Sciences*, *9*(5), 84.

Chang, S. H., & Bhat, C. S. (2023). A group counseling model based on Bowen Family Systems Theory for college students with codependency in Taiwan. *The Journal for Specialists in Group Work*, *48*(3), 196-211.

Çimşir, E., & Akdoğan, R. (2021). Childhood Emotional Incest Scale (CEIS): Development, validation, cross-validation, and reliability. *Journal of counseling psychology*, *68*(1), 98.

Costa, S., Barberis, N., Griffiths, M. D., Benedetto, L., & Ingrassia, M. (2021). The love addiction inventory: Preliminary findings of the development process and psychometric characteristics. *International Journal of Mental Health and Addiction*, *19*, 651-668.

Costa, C. M. R. F. D., & Oliveira-Monteiro, N. R. D. (2020). Codependency, psychological problems and time of exposure to parents with a history of psychoactive substance dependence: appointments. *Contextos Clínicos*, *13*(3), 724-739.

Danilov, D., & Walsh, S. D. (2023). Fragile functionality: The experience of immigrant young adults in Israel who grew up with a parent addicted to alcohol. Children and Youth Services Review, 152, 107062.

Dineen, J., & Dinc, L. (2024). Love addiction: Trait impulsivity, emotional dysregulation and attachment style. *The European Journal of Psychiatry*, *38*(3), 100255.

Diotaiuti, P., Mancone, S., Corrado, S., De Risio, A., Cavicchiolo, E., Girelli, L., & Chirico, A. (2022). Internet addiction in young adults: the role of impulsivity and codependency. *Frontiers in psychiatry*, *13*, 893861.

Earp, B. D., Wudarczyk, O. A., Foddy, B., & Savulescu, J. (2017). Addicted to love: What is love addiction and when should it be treated?. *Philosophy, Psychiatry, & Psychology*, *24*(1), 77-92.

Ermakov, P. N., Butenko, V. S., Kolenova, A. S., & Saakyan, O. S. (2019). Diagnostic possibilities of a method of content analysis for therapy of women exhibiting codependency. In *SHS Web of Conferences* (Vol. 70, p. 09003). EDP Sciences.

Escuza Pasco, M. E., & Chunga Diaz, T. O. (2022). Efficacy of an intervention program for co-dependent family members of institutionalized drug addicts. *NeuroQuantology*, *20*(8), 6541-6552.

Esmaeilpour, R. (2024). *Women’s Perceptions of How Sexual Trauma Impacts Intimate Bonds in Recovery*. California State University, Long Beach.

Fard, J. H., Mirzaian, B., & Hoseini, S. H. (2019). Craving and psychological injury, with the mediating role of codependency and self-control in patients undergoing maintenance therapy. *Journal of Nursing and Midwifery Sciences*, *6*(3), 118-124.

Fernandez, D. P., Kuss, D. J., & Griffiths, M. D. (2021). Lived experiences of recovery from compulsive sexual behavior among members of sex and Love Addicts anonymous: A qualitative thematic analysis. *Sexual Health & Compulsivity*, *28*(1-2), 47-80.

Giacobbe, C., Maggi, G., Borrello, L., Barone, A., Mastromarino, C., Antonelli, P., & Santangelo, G. (2024). Psychological and cognitive complaints in individuals with love addiction. *Journal of Affective Disorders Reports*, *16*, 100785.

Gomberg, E. S. L. (2019). On terms used and abused: the concept of “codependency”. In *Current issues in alcohol/drug studies* (pp. 113-132). Routledge.

Gori, A., Russo, S., & Topino, E. (2023). Love addiction, adult attachment patterns and self-esteem: Testing for mediation using path analysis. *Journal of Personalized Medicine*, *13*(2), 247.

Gori, A., Topino, E., Russo, S., & Griffiths, M. D. (2024). A pilot study on childhood trauma and love addiction: Exploring the mediation of unbalanced family functioning. *Psychological Trauma: Theory, Research, Practice, and Policy*.

Graham, C. (2022). *Demystifying Toxic Romantic Relationships: Identifying Behaviors and Post-breakup Outcomes* (Doctoral dissertation, Arizona State University).

Grier, S. C. (2022). Capturing interpersonal and momentary dynamics of codependency (Doctoral dissertation, Long Island University). Digital Commons @ LIU. https://digitalcommons.liu.edu/post_fultext_dis/434

GRIGORIEVA, L. M. (2024). SELF-IMAGE OF WOMEN IN CO-DEPENDENCE: A PSYCHOLINGUISTIC APPROACH. *Экспериментальная психология*, *17*(3), 159.

Gunko, B., Viltsaniuk, O., & Stepaniuk, A. (2019). THE PROBLEM OF CO-DEPENDENCE AMONG HIV-INFECTED DRUG-ADDICTED FAMILY MEMBERS. *Georgian Medical News*, (286), 87-90.

Hall, P. (2013). A new classification model for sex addiction. *Sexual Addiction & Compulsivity*, *20*(4), 279-291.

Hentsch-Cowles, G., & Brock, L. J. (2013). A systemic review of the literature on the role of the partner of the sex addict, treatment models, and a call for research for systems theory model in treating the partner. *Sexual Addiction & Compulsivity*, *20*(4), 323-335.

Hill, L. A. (2023). *Addicted to Love: Exploring an Attachment Approach to Treat Addiction in Couples Therapy*. The Chicago School of Professional Psychology.

Hodge, J. (2016). *Adult children of alcoholics and the effects on relationships*. Kean University.

Kaur, S. (2016). A descriptive study to assess depression and codependency among wives of alcoholics in a selected rural community of Gurdaspur, Punjab. *Asian Journal of Nursing Education and Research*, *6*(2), 183-187.

Kemer, G., Yıldız, E. Ç., & Bulgan, G. (2016). Emotional dependency and dysfunctional relationship beliefs as predictors of married Turkish individuals’ relationship satisfaction. *The spanish journal of psychology*, *19*, E72.

Kim, S. H., Baek, M., & Park, S. (2021). Association of parent–child experiences with insecure attachment in adulthood: A systematic review and meta‐analysis. *Journal of Family Theory & Review*, *13*(1), 58-76.

Knapek, E., & Szabó, K. (2014). The concept, the symptoms and the etiological factors of codependency. Psychiatria Hungarica: A Magyar Pszichiatriai Tarsasag Tudomanyos Folyoirata, 29(1), 56-64.

Kolenova, A., Denisova, E., Kukulyar, A., & Ermakov, P. (2023). Personality traits, approval motivation, and empathy as predictors of cognitive regulation of emotions and behavioral self‐control in codependent women. *International Journal of Cognitive Research in Science, Engineering and Education*, *11*(2), 187–197. <https://doi.org/10.23947/2334-8496-2023-11-2-187-197> [ijcrsee.com+9doaj.org+9grafiati.com+9](https://doaj.org/article/ba1f32b66e784e7985b25b47db9f2dba?utm_source=chatgpt.com)

Kolenova, A., Kukulyar, A., Brizhak, Z., & Bessonova, N. (2021). The concept of success in representing codependent women: transformation in the conditions of the COVID-19 pandemic threat. In *E3S Web of Conferences* (Vol. 273, p. 10034). EDP Sciences.

Kornaszewska-Polak, M. (2019). Mothers’ co-dependence and their daughters’ patterns of attachment and romantic relationships as adult children of alcoholics: an explorative study. *Alcoholism and Drug Addiction/Alkoholizm i Narkomania*, *32*(3), 153-174.

Lee, G., & Kim, S. (2016). The effects of an enneagram program on codependency, anger, and interpersonal relationship in wives of Alcoholics. *Journal of Korean Academy of Psychiatric and Mental Health Nursing*, *25*(3), 166-175.

Lemos, M., & Román-Calderón, J. P. (2019). Potential therapeutic targets in people with emotional dependency. International Journal of Psychological Research, 12(1), 18-27.

Lymankina, A. (2023). Psychological features of women's codependency during the midlife crisis. *Sci Bull Mukach State Univer Ser Ped Psychol*, *9*, 80-86.

Maglia, M. G., Lanzafame, I., Quattropani, M. C., & Caponnetto, P. (2023). Love addiction-current diagnostic and therapeutic paradigms in clinical psychology. *Health psychology research*, *11*, 70218.

Maina, G., Ogenchuk, M., Phaneuf, T., & Kwame, A. (2021). “I can’t live like that”: the experience of caregiver stress of caring for a relative with substance use disorder. *Substance Abuse Treatment, Prevention, and Policy*, *16*, 1-9.

Malagayo, N. (2024). The influence of parentification on mental health help-seeking behaviours: A phenomenological study (Doctoral dissertation, Doctoral thesis, University of Canterbury).

Margasiński, A. (2022). Dysfunctional symptom intensity in wives of alcoholics as measured by the Emotional Dependency Questionnaire (EDQ). *Alcoholism and Drug Addiction/Alkoholizm i Narkomania*, *35*(1), 43-66.

Mashuri, M. F., & Patriasih, A. I. (2023). Bucin (Budak Cinta): The Other Side of Love Addiction in Romantic Relationships in Indonesia. *Journal An-Nafs: Kajian Penelitian Psikologi*, *8*(1), 1-19.

Momeñe, J., Estévez, A., Etxaburu, N., Pérez-García, A. M., & Maguregi, A. (2022). Emotional dependence on the aggressor partner and its relationship to social anxiety, fear of negative evaluation and dysfunctional perfectionism. *Behavioral Psychology= Psicología Conductual*, *30*(1), 51-68.

Navarro, G. H. (2023). *The Ambiguous Loss of Alcohol Use Disorders for Affected Family Members: Can Al-Anon Involvement and Psychological Flexibility Make a Difference?*. Northern Illinois University.

Odokonyero, R., Aujo, T., Agaba, D., & Abbo, C. (2022). Childhood adversity and co-dependency roles in a case of a midwife with pethidine use disorder attending Mulago National Referral Hospital, Kampala, Uganda. *Cogent Public Health*, *9*(1), 2145704.

Orsolini, S., Antonelli, P., Salvatori, G., & Dèttore, D. (2022). Suicidal ideation in a group of Italian love addicts: a qualitative research. *Sexual Health & Compulsivity*, *29*(1-2), 56-67.

Özal, Z., Mancini, G., De Fino, G., Ambrosini, F., Biolcati, R., & Truzoli, R. (2023). “I Can’t Do without You”: Treatment Perspectives for Affective Dependence: A Scoping Review. *Journal of Clinical Medicine*, *12*(21), 6769.

Pant, S., Mishra, S., & Kar, S. K. (2022). Significant co-dependency, anxiety, depression and family burden among the caregivers of patients with opioid dependence syndrome: an observational study. *Global Psychiatry Archives*, *5*(2), 90-104.

Pekyiğit, A., Yıldırım, T., & Bağrıyanık, B. Ç. (2024). Examining the relationship between pediatric nurses’ liking of children levels and their codependency. *Nursing Practice Today*, *11*(1), 64-71.

Petruccelli, F., Diotaiuti, P., Verrastro, V., Petruccelli, I., Federico, R., Martinotti, G., ... & Janiri, L. (2014). Affective dependence and aggression: An exploratory study. *BioMed Research International*, *2014*(1), 805469.

Pugliese, E., Saliani, A. M., Mosca, O., Maricchiolo, F., & Mancini, F. (2023). When the War Is in Your Room: A Cognitive Model of Pathological Affective Dependence (PAD) and Intimate Partner Violence (IPV). *Sustainability*, *15*(2), 1624.

Redcay, A., & McMahon, S. (2021). Assessment of relationship addiction. *Sexual and Relationship Therapy*, *36*(1), 116-125.

Rhodes, T., Rance, J., Fraser, S., & Treloar, C. (2017). The intimate relationship as a site of social protection: Partnerships between people who inject drugs. *Social Science & Medicine*, *180*, 125-134.

Rice, J. S. (2017). *A disease of one's own: Psychotherapy, addiction and the emergence of co-dependency*. Routledge.

Roberts, G. D. (2016). *Developmental Traits and Patterns Emerging from Dependent Nurturing Individuals in Narcissistic Relationship*. University of Arkansas.

Roberts, M. D. (2021). *Moth to a Flame: an Investigation of the Personality Traits and Early-Life Trauma Histories of Women Who Have Survived Adult Relationships with Men with Pathological Narcissism*. University of Missouri-Saint Louis.

Rodríguez Blanco, A. E. (2013). Resilient women: from victimhood to autonomy case study in the self-help groups codependent anonymous. *Acta colombiana de psicología*, *16*(2), 71-79.

Ruiz-García, A., Jiménez, Ó., Resurrección, D. M., Ferreira, M., Reis-Jorge, J., & Fenollar-Cortés, J. (2021). Portuguese validation of the Adult Separation Anxiety—Questionnaire (ASA-27). *Plos one*, *16*(3), e0248149. Ruiz-García, A., Jiménez, Ó., Resurrección, D. M., Ferreira, M., Reis-Jorge, J., & Fenollar-Cortés, J. (2021). Portuguese validation of the Adult Separation Anxiety—Questionnaire (ASA-27). *Plos one*, *16*(3), e0248149.

Sanches, M., & John, V. P. (2019). Treatment of love addiction: Current status and perspectives. The European Journal of Psychiatry, 33(1), 38-44.

Salani, A., Antonelli, P., Salvatori, G., Gritti, M. C., Bisciglia, R., Mascherini, F., & Dèttore, D. (2022). Love addiction, emotional dysregulation and attachment bonds: A quantitative study of 344 females. *Sexual Health & Compulsivity*, *29*(3-4), 127-148.

Sirvent-Ruiz, C. M., Moral-Jiménez, M. D. L. V., Herrero, J., Miranda-Rovés, M., & Rodríguez Díaz, F. J. (2022). Concept of affective dependence and validation of an Affective Dependence Scale. *Psychology Research and Behavior Management*, 3875-3888.

Spivakovskaya, A. S., & Lutsenko, A. M. (2021). Resource Factors Allowing People with Alcohol-addicted Parents to Overcome Their Negative Emotions: A Latent Variable Model and Content Analysis. *Psychology in Russia*, *14*(2), 25.

Tekin, H., & Ançel, G. (2023). Codependency, gender equality, and sociodemographic variables as predictors of psychological well-being in homemakers. *Journal of Psychiatric Nursing*, *14*(4).

Turan, N., Ancel, G., & Canbulat, Ş. (2021). Examination of student nurses' self-recognition and codependence. *Journal of Psychiatric Nursing*.

Ulusoy, Y., & Güçray, S. S. (2019). Effect of Encounter Group Application Integrated with Psychodrama Techniques on Codependency. *Current Approaches in Psychiatry/Psikiyatride Guncel Yaklasimlar*, *11*.

Vasilyeva, S. (2023). *Interpretive Phenomenological Analysis of Holotropic Breathwork for Adult Daughters of Alcoholics*. Pacifica Graduate Institute.

Walters, K. J., & Simons, J. S. (2023). Affective dependence, self-regulation, and alcohol problems: Between-and within-person associations. *Emotion*, *23*(8), 2142.

Weegmann, M., & Head, A. (2016). Circles of care: Applied group analysis with family and friends. *Group Analysis*, *49*(4), 431-451.

Weiss, R. (2018). *Prodependence: Moving beyond codependency*. Simon and Schuster.

Weiss, R. (2022). *Prodependence: Moving Beyond Codependency: Revised Edition*. Simon and Schuster.

Woolfolk, A. (2017). The dubious triumph of the therapeutic: The denial of character. In *Therapeutic Culture* (pp. 69-88). Routledge.

Wnuk, M. (2015). Religious–spiritual sources of hope and the meaning of life in alcohol co-dependent subjects receiving support in self-help groups. *Journal of Substance Use*, *20*(3), 194-199.

Wnuk, M. (2023). The mechanism underlying the relationship between the spiritual struggles and life satisfaction of Polish codependent individuals participating in Al-Anon–pilot study. *Journal of Spirituality in Mental Health*, *25*(3), 198-218.

Wright, N. M., Dmitrieva, J., & DePrince, A. P. (2021). Dependence in adult relationships: Latent classes of relational dependence and associated outcomes in women exposed to intimate partner abuse. *Psychological trauma: theory, research, practice, and policy*, *13*(3), 359.

Yang, S., & Hong, J. A. (2015). Factors influencing family-function in families of pathological gamblers. *Journal of Korean Academy of Psychiatric and Mental Health Nursing*, *24*(3), 196-206.

Young, L. B., & Timko, C. (2015). Benefits and costs of alcoholic relationships and recovery through Al-Anon. *Substance use & misuse*, *50*(1), 62-71.

Yoshii, H. (2023). A Conceptual Analysis of Recovery of Family Members in Codependent Relationships With People With Addiction Problems. *Journal of Japan Academy of Nursing Science*, *43*.

Zaidi, U. (2015). Co-dependency and relationship satisfaction among spouses of alcohol abusers. *Journal Of Humanities And Social Science (IOSR-JHSS)*, *20*(1), 86-91.

Zerbetto, S. R., Galera, S. A. F., & Ruiz, B. O. (2017). Family resilience and chemical dependency: perception of mental health professionals. Revista brasileira de enfermagem, 70(6),

**Supplementary File 3. Extraction Tables**

**Table C1**

| *Characteristics of Quantitative Studies* | |
| --- | --- |
| **Atintaş & Tutarel-Kışlak, 2019**  ***Turkey*** |  |
| Type | Correlational and comparative |
| Study Aim | To compare marital adjustment and power, co-dependency, depression, anxiety, and stress in wives. |
| Population | Wives of alcoholics whose partner was undergoing treatment in 3 different centres in Turkey and a comparison group. Sample size: 100; Gender: F; Age: M=41.17 years. (SD=9.47) |
| Method | Sampling: purposive for the experimental group, convenience for the comparison. Data collection: The Marital Adjustment Test, Codependency Assessment Tool (CODAT), Depression-Anxiety-Stress Scale, Couple Power Scale. Analysis: independent samples t-test and regression. |
| Co-dependency definition | A phenomenon characterised by other-focus/self-neglect with four sub-concepts (low self-worth, hiding self, psychosomatic problems, and family-of-origin issues). |
| Framework | Socio-cultural and social psychology perspective. |
| Findings | Conceptualisation: Co-dependency is higher in wives of alcoholics; MH outcomes: Co-dependency negatively correlated with marital adjustment, power, and life satisfaction, and positively correlated with depression, anxiety, stress. |
| **Bespalov et al., 2024**  ***Turkey*** |  |
| Type | Correlational and comparative |
| Study Aim | To determine the individual psychological characteristics of men and women in codependent marital relationships. |
| Population | Couples who sought psychological counselling for co-dependency; Sample size: 85; Gender: 46 F, 39 M; Age: M=32.75 years (SD not reported). |
| Method | Sampling: random purposive; Data collection: The Co-dependency Self- Inventory Scale (CSIS); Scale for measuring the level of co-dependency, Interpersonal Dependency Inventory; Test-questionnaire for determining self-esteem; Diagnosis of emotional intelligence; Coping test. Analysis: student t-test, ANOVA, correlation. |
| Co-dependency definition | A socio-psychological phenomenon involving emotional interdependence, low self-worth and autonomy, particularly in the context of marital relationships. |
| Framework | Attachment theory; Family system theory; Emotional regulation theories; Socio-cultural/social psychology perspective. |
| Findings | The formation of codependent relationships is influenced by low self-esteem. Codependent men and women showed low self-esteem and emotional intelligence. Women tend to adapt to codependent relationships due to low emotional management and higher empathy, while men cope through confrontation and distancing. |
| **Chang, 2018**  ***Taiwan*** |  |
| Type | Correlational |
| Study Aim | To test a model of co-dependency based on Bowen’s concept of differentiation. |
| Population | College students in Taiwan. Sample size: 576; Gender: 372 F, 195 M; Age: M=20.44(SD=1.86). |
| Method | Sampling: Convenience; Data collection: CODAT, Chinese Version of the Differentiation of the Self Inventory (DSI-R). Family Assessment Device (FAD), Experiences in Close Relationships Scale-Chinese version, Rosenberg’s Self-Esteem Scale (RSE), General Health Questionnaire. Analysis: Pearson correlation and SEM. |
| Co-dependency definition | A complex construct characterized by low self-differentiation, other-focused behaviours, and relationship anxiety, emerging from family-of-origin dysfunction. |
| Framework | Attachment theory; Family system theory; Cultural perspective. |
| Findings | Co-dependency is linked to social dysfunctions and impaired psychological adjustment. Lower self-differentiation partially mediates the effect of family-of-origin dysfunction on co-dependency. |
| **Eshan & Suneel, 2020**  ***Pakistan*** |  |
| Type | Correlational |
| Study Aim | To investigate the relationship between co-dependency and mental health functioning with relation to gender of parents with intellectually disabled children. |
| Population | Sample size: 41; Gender: 20 F, 21 M; Age: M=35.98(SD=7.19). |
| Method | Sampling: random-based convenience; Data collection: The Depression-Stress-Anxiety Scale-21, Spann-Fischer co-dependency scale (SF-CDS). Analysis: Pearson correlation and ANCOVA. |
| Co-dependency definition | Dysfunctional ways of relating, can develop in response to caregiving burden. |
| Framework | Not reported. Aligns with system theory and stress-coping perspective. |
| Findings | MH outcomes: Co-dependency negatively correlated with mental health functioning. Gender did not significantly predict mental health when co-dependency was controlled for. |
| **Evgin & Sümen, 2022**  ***Turkey*** |  |
| Type | Descriptive and correlational |
| Study Aim | To determine the relationship between childhood neglect and abuse and co-dependency, and the factors affecting co-dependency. |
| Population | Nursing and child development students. Sample size: 292; Gender: 207 F, 85 M; Age: M=20.25 (SD=1.27). |
| Method | Sampling: random-based convenience. Data collection: CODAT, the Childhood Trauma Questionnaire (CTQ), RSE, the Beck Depression Inventory, the Styles of Coping with Stress Scale. Analysis: Mann–Whitney U test, Kruskal–Wallis H test, Spearman's correlation. |
| Co-dependency definition | A psychosocial issue involving maladaptive coping mechanisms and disrupted self-worth, shaped by early relational experiences and continued through adult relationships, particularly in caregiving contexts. |
| Framework | Developmental perspective; Stress-strain theory; Sociocultural perspective; Attachment theory; Family system theory. |
| Findings | Conceptualisation: There is a positive relationship between childhood neglect and abuse and co-dependency. MH outcomes: A negative relationship was found between co-dependency and self-esteem, depression, and stress-coping. |
| **Happ et al., 2023**  ***Hungary*** |  |
| Type | Correlational |
| Study Aim | To examine how co-dependency influences negative dyadic coping, perceptions of relationship problems, and affects life satisfaction through these factors. |
| Population | Hungarian adults in a relationship. Sample size: 246; Gender: 167 F, 79 M; Age: 18-72 years, M=35.03(SD=11.6). |
| Method | Sampling: convenience; Data collection: SF-CDS, the Dyadic Coping Inventory, the Shortened Marital Stress Scale, the Satisfaction With Life Scale. Analysis: Pearson correlation and SEM. |
| Co-dependency definition | A stable attitude that determine a person’s perception and behaviour, manifesting in dysfunctional pattern of relating to others. |
| Framework | Personality psychology; Family systems theory. |
| Findings | Conceptualisation: Codependency is associated with negative dyadic coping and perception of relationship problems. MH outcomes: Co-dependency, negative dyadic coping and relationship problems perception predicted lower life satisfaction. |
| **Hawkins & Hawkins, 2014**  ***USA*** |  |
| Type | Correlational and comparative |
| Study Aim | To explore the relationship between co-dependency, gender, positive and negative gender-stereotyped traits, and other measures of personality and problem drinking. |
| Population | American social work undergraduates. Sample size: 208; Gender: 167 F, 41 M, 2 unspecified; Age: M=23.6 years (SD=5.6). |
| Method | Sampling strategy: convenience. Data collection: Beck's Codependent Assessment Scale, the ACOA Tool, the Internalized Shame Scale, The Drinking Restraint Scale, the Personal Style Inventory, the Sensation Seeking Scale, Short Michigan Alcoholism Screening Test, the Extended Personal Attributes Scale. Analysis: MANOVA, ANOVA, regression. |
| Co-dependency definition | A dimension of personality, varying by degree from normality to deviance, as operationalised by gender-stereotyped attributes, which may be expressed by both women and men. |
| Framework | Personality and social psychology perspectives. |
| Findings | Co-dependence does not differ by gender and is more prevalent among students with a positive family history of alcohol problems. It is negatively correlated with socially desirable masculinity and femininity traits and is linked to ACOA traits, shame, and vulnerability to depression. Contradependence is associated with sensation seeking, negative masculinity, and problem drinking tendencies. |
| **Kaplan, 2023**  ***Turkey*** |  |
| Type | Correlational and descriptive |
| Study Aim | To examine the mental health states of housewives within the framework of co-dependence and self-perceptions. |
| Population | Housewives in Turkey. Sample size: 371; Gender: F; Age: M=35.19(SD=9.85). |
| Method | Sampling: snowballing; Data collection: CODAT. social comparison scale, the Symptom Checklist-90-Revised. Analysis: observed variable path analysis, SEM. |
| Co-dependency definition | A characteristic that develops in dysfunctional families, associated with self-neglecting, other-focus and emotional suppression. |
| Framework | Socio-psychological gender perspective; Psychoanalytic perspective. |
| Findings | MH outcomes: mental health correlated with co-dependency and self-perceptions. Higher co-dependency and negative self-perception increased the psychological symptoms. |
| **Karaşar, 2020**  ***Turkey*** |  |
| Type | Correlational |
| Study Aim | To test the mediator role of the need for social approval in the relationship between perfectionism and co-dependency. |
| Population | Pre-teachers in Turkey; Sample size: 188; Gender: 144 F and 44 M; Age: not reported. |
| Method | Sampling: random-based convenience. Data collection: SF-CDS, Need for Social Approval Scale and Frost Multidimensional Perfectionism Scale. Analysis: SEM. |
| Co-dependency definition | A pattern of self-neglect, driven by social approval need, perfectionism, and cultural expectations of self-sacrifice, leading individuals to prioritise others' needs over their own. |
| Framework | Schema therapy model; Social psychology perspective; Cultural perspective. |
| Findings | Conceptualisation: Social approval plays a partial mediating role in the relationship between perfectionism and co-dependency, suggesting that the need for social validation is a key factor linking perfectionistic tendencies to co-dependency. |
| **Kaya et al., 2024**  ***Turkey*** |  |
| Type | Correlational |
| Study Aim | To investigate the mediating role of resilience in the relationship between childhood emotional abuse and emotional neglect and co-dependency in young adults. |
| Population | Turkish young adults in different life stages; Sample size: 401;  Gender: 305 F and 96 M; Age: M=35.6 (SD not reported). |
| Method | Sampling: convenience; Data collection: SF-CDS, the Emotional Abuse and Emotional Neglect subscales of the CTQ, the Adult Resilience Measure; Analysis: regression. |
| Co-dependency definition | Relationship addiction: pathological condition characterised by overreliance on interpersonal relationships. |
| Framework | Attachment theory, developmental psychology, resilience perspective. |
| Findings | Childhood emotional abuse and neglect contribute to co-dependency. Resilience partially mediates the relationship between abuse and co-dependency but not neglect. |
| **Knapek et al., 2017**  ***Hungary*** |  |
| Type | Comparative |
| Study Aim | To identify whether ‘pure’ codependent individuals exist. Pure co-dependency refers to the condition of codependent individuals without BPD and/or DPD. |
| Population | Hungarian adults engaging with psychiatry, self-help groups or from the general population. Sample size: 407; Gender: 335 F, 72 M; Age: 18- 70 years. |
| Method | Sampling: convenience; Data collection: The Structured Clinical Interview for DSM-IV Axis 11 Personality Disorders (SCID-II), the Co-dependent Questionnaire (CdQ). Analysis: Chi-square tests. |
| Co-dependency definition | A mental problem characterised by extreme caretaking, enabling behaviour, and responsibility for others. |
| Framework | Disease model; PD Perspective |
| Findings | Borderline and dependent traits are common among co-dependents. However, 16% of co-dependents do not display these traits, suggesting that co-dependency can exist as a distinct concept separate from PD. |
| **Knapek et al., 2021**  ***Hungary*** |  |
| Type | Predictive |
| Study Aim | To identify the factors best able to predict co-dependency while controlling for BPD and DPD traits. |
| Population | Hungarian adults engaging with psychiatry, self-help groups or from the general population. Sample size: 192; Gender: 143 F,49 M; Age: 18-45. |
| Method | Sampling: convenience; Data collection: SCID-II, CdQ, the Traumatic Antecedents Questionnaire, the Young Schema Questionnaire, the Parentification Questionnaire - Adult. Analysis: Linear multiple regression. |
| Co-dependency definition | A behavioural addiction which can play a role in maintaining others’ addictive behaviours. |
| Framework | Disease & addiction model; Schema Therapy; Family system theory. Behavioural addiction framework. |
| Findings | Co-dependency is predicted by subjugation and self-sacrifice schemas, mental disorder diagnosis, female gender, borderline traits and parentification. |
| **Lampis et al., 2017**  ***Italy*** |  |
| Type | Correlational/predictive |
| Study Aim | To assess the validity of a model in which codependent behaviours were predicted by self-differentiation and dyadic adjustment. |
| Population | Adults in a relationship living in Italy. Sample size: 318; Gender: 160 F, 158 M; Age: 19- 81 years, M=47.32 (SD=15.7). |
| Method | Sampling: convenience; Data collection: DSI-R, the Dyadic Adjustment Scale, CSIS. Analysis: Pearson’s correlation, independent t-test, multiple linear regression. |
| Co-dependency definition | An affective disorder developing from the internalisation of family-of-origin experiences. It manifests as a relationship addiction. |
| Framework | Family Systems Theory; Cultural perspective; Behavioural addiction framework. |
| Findings | The dimensions of self-differentiation were more important in explaining co-dependency compared to dyadic adjustment. The most important variables in predicting co-dependency were emotional reactivity and emotional cutoff. |
| **Rozhnova et al., 2020**  ***Russia*** |  |
| Type | Case-control |
| Study Aim | To study the psychological and genetic components of co-dependency. |
| Population | Three groups of Russian women: (1) co-dependents, 2) phenotypically healthy; 3) general population. Sample size: 256; Gender: F; Age: M=46.4 (SD=11.8). |
| Method | Sampling: purposive; Data collection: 1) IC10 Clinical interview. 2) The Codependency Scale, questions for self-diagnosis, The «hand test» 3) Clinical and genealogical testing. Analysis: ANOVA, Student’s t-test, Chi-square. |
| Co-dependency definition | An addictive behaviour disorder influenced by early family dynamics, unmet needs, and dysfunctional relationships. |
| Framework | Biopsychosocial perspective; Behavioural addiction framework. |
| Findings | Conceptualisation: Codependency has psychological and genetic components. Codependent women showed auto aggressive behaviours and a family history of alcoholism. MH outcomes: risk of mental and physical health issues, psycho-emotional overstrain, somatoform disorders. |
| **Tunca et al., 2024**  ***Turkey*** |  |
| Type | Correlational and comparative |
| Study Aim | To compare the co-dependency characteristics of individuals with and without dependent relatives, focusing on personal (defense mechanisms), domestic (family functionality), and relational (attachment) contexts. |
| Population | Clinical group recruited from a substances treatment centre in Turkey. Non-clinical group recruited via social media. Sample size: 115; Gender: 71.3% F, 28.7% M; Age: 19-69 years, M=40.88 years, (SD=12.56). |
| Method | Sampling: purposive; Data collection: CODAT, Defense Styles Questionnaire, FAD, Relationship Scales Questionnaire. Analysis: Independent t-tests, Pearson correlations, Hierarchical multiple linear regression. |
| Co-dependency definition | A condition involving psychopathology, dysfunctional family systems, and maladaptive relational patterns. It manifests as self-neglect, low self-worth, and preoccupied attachment, with an overemphasis on others. |
| Framework | Family Systems Theory; Psychoanalytic theory; Attachment theory. |
| Findings | Conceptualisation: Codependency is influenced by defences, family dysfunction and preoccupied attachment. |
| **Vederhus et al., 2019**  ***Norway*** |  |
| Type | Validation and correlational study |
| Study Aim | To validate the Composite Codependency Scale (CCS). To investigate the relationship between co-dependency and family functioning and co-dependency and quality of life. |
| Population | CSO of patients in treatment for SUD and a control group. Sample size: 664; Gender: 479 F, 185 M; Age: M=44.5 (SD not reported). |
| Method | Sampling: convenience; Data collection: CCS, the general family functioning subscale from the McMaster Family Assessment Device, the Quality-of-Life Scale. Analysis: CFA, latent regression model. |
| Co-dependency definition | A phenomenon characterised by self-sacrifice, interpersonal control, and emotional suppression. |
| Framework | Addiction and family system theories. |
| Findings | Conceptualisation: Family members of individuals with SUD exhibit higher co-dependency, characterised by greater emotional suppression and interpersonal control. MH outcomes: Co-dependency was associated with greater family dysfunction and worse quality of life. |
| **Zielinski et al., 2019**  ***USA*** |  |
| Type | Quasi-experimental with correlational and comparative elements. |
| Study Aim | To examine associations between co-dependency and brain functioning. |
| Population | CSO of individuals with SUD and a control group; Sample size: 38; Gender: 30 F,8 M; Age: M=37.41 (SD=14.19). |
| Method | Sampling: purposive; Data collection: functional near‐infrared spectroscopy while participants viewed images of a loved‐one with SUD or of a “target family member”. SF-CDS. Data analysis: FnIR processing , t-test and bivariate correlations. |
| Co-dependency definition | A learned dysfunctional condition, manifesting as excessive focus on a loved‐one with SUD despite negative consequences. |
| Framework | Addiction models; Family system theory; Biopsychosocial perspective. |
| Findings | Conceptualisation: Co-dependency is negatively associated with left dorsomedial PFC activation in response to images of a loved one with SUD, MH outcomes: Brain activation suggests that co-dependency may impair the ability to effectively regulate emotions in response to relationship stress. |

**Table C2**

| *Characteristics of Qualitative Studies* | |
| --- | --- |
| **Aristizábal, 2020**  ***Columbia*** |  |
| Study Aim | To explore co-dependency in the relationships of imprisoned women. To investigate the relationship between co-dependency and violent crimes. |
| Population | Women reporting a romantic bond pre or during imprisonment. Sample size: 27, Gender: F, Age: Not reported. |
| Method | Sampling strategy: purposive; Data collection: in-depth interviews, focus groups and the Emotional Co-dependency Inventory. Data analysis: Descriptive and discourse analysis |
| Co-dependency definition | Emotional dependency characterised by control and enabling behaviours. |
| Framework | Psycho-social perspective; Socio-constructionist perspective. |
| Findings | Conceptualisation: Co-dependency is influenced by gender roles, leading to behaviours such as denial, incomplete identity, repression, and rescuing; MH outcomes: Co-dependency contributed to crime involvement, distress and relationship challenges as reflected in themes: 1) I did it for him 2) Although he doesn’t love me and 3) I preferred to remain silent. |
| **Bacon et al., 2020**  ***UK*** |  |
| Study Aim | To explore the lived experiences of co-dependency. |
| Population | CoDA fellows; Sample size: 8; Gender: 5 F, 3 M; Age: mid-30s to mid-60s. |
| Method | Sampling strategy: purposive; Data collection: in-depth semi structured interviews and a visual method; Data analysis: IPA. |
| Co-dependency definition | A complex, psychosocial problem, seen as both an adaptive coping strategy and a socially accepted form of addiction. |
| Framework | Family system theory; Psychoanalytical perspective; Developmental perspective. |
| Findings | Conceptualisation: Participant conceptualised co-dependency as manifesting through emotional instability and an unclear sense of self and resulting from difficult childhood experiences. |
| **Klimczak & Kiejna, 2018**  ***Poland*** |  |
| Study Aim | To explore the biographies of co-dependent women. To understand the relationship between their significant life events and the process of creating beliefs about themselves, interpreting and giving their own lives a meaning, and how they shape relationships with others. |
| Population | Polish co-dependent women receiving psychological support. Sample size: 32, Gender: F. Age: age range 28-68 years; Mean age 47 (SD not reported) |
| Method | Sampling strategy: purposive; Data collection: semi-structured narrative autobiographical interviews, with timeline drawing method. Data analysis: Narrative and thematic content analysis applying the Big five. |
| Co-dependency definition | Adaptive response to stress and relational trauma, particularly in dysfunctional family settings. |
| Framework | Personality psychology; Developmental perspective; Stress perspective. |
| Findings | Conceptualisation: Co-dependent behaviours are a manifestation of childhood trauma. Co-dependency is associated to high levels of neuroticism and conscientiousness, a moderate level of agreeableness, and low levels of openness to experiences and extroversion. |
| **Nordgren et al., 2020**  ***Sweden*** |  |
| Study Aim | To analyse how parents of adult children with drug problems talked about and understand co-dependency. |
| Population | Swedish parents. Sample size: 32, Gender: 24 F, 8 M, Age: 46-70 years. |
| Method | Sampling strategy: purposive; Data collection: semi-structured interviews; Data analysis: TA. |
| Co-dependency definition | A range of behaviours shaped by societal expectations among individuals who are affected by the drug use of family members. |
| Framework | Social constructionist & social psychology perspective. |
| Findings | Conceptualisation: Co-dependency appears to be more of an externally attributed label than an internally recognized identity, at least initially.  MH outcomes: Participants faced distress due to co-dependency, experiencing guilt and ambivalence between supporting their children and setting boundaries, as a response to reconcile societal expectations. |
| **Sobol-Goldberg et al., 2024**  ***Israel*** |  |
| Study Aim | To explore the perceptions, lived experiences, and coping of women who live with spouses who have SUD in response to implicit and explicit messages from professionals and others in their environment. |
| Population | Women treated in out to five outpatient treatment centres in Israel. Sample size: 12, Gender: F, Age: 30-69 years; Mean age 46 (SD not reported) |
| Method | Sampling strategy: purposive; Data collection: semi-structured interviews; Data analysis: Content analysis. |
| Co-dependency definition | A relational phenomenon that has negative connotations and may affect the way people and society relate to family members of individuals with addiction. |
| Framework | Critical & social constructionist lens; Attachment models; Psychoanalytic perspective; Family System theory. |
| Findings | MH outcomes: Women internalised three types of social messages which impacted on their wellbeing: 1)Messages leading to guilt, shame, and self-stigma, 2)Messages contributing to exclusion and isolation, 3)Messages supporting their caregiving role, which sometimes strengthened their sense of value. |

**Table C3**

| *Characteristics of Conceptual Papers* | | | |
| --- | --- | --- | --- |
| **Bacon & Conway, 2023**  ***UK*** | | |  |
| Type | | Commentary | |
| Study Aim | | To explore the conceptual overlap between co-dependency and enmeshment. To introduce the CODEM model for practical application. | |
| Method | | Literature Review & Case Illustration | |
| Co-dependency definition | | A complex condition rooted in early family dynamics and unmet needs and involving maladaptive schemas. | |
| Framework | | Schema therapy; Family system theory. | |
| Findings | | Co-dependency was shown to be an outward manifestation of enmeshment, characterised by impaired autonomy and self-sacrifice. | |
| **Calderwood & Rajesparam, 2014**  ***Canada*** |  | |  |
| Type | Commentary | |  |
| Study Aim | To provide a critique of the application of the co-dependency framework in gamblers. | |  |
| Population | N/A | |  |
| Method | Critical analysis | |  |
| Co-dependency definition | A stigmatising term describing COS as having dysfunctional traits. | |  |
| Framework | Stress-coping perspective. | |  |
| *Findings* | There is no evidence that the co-dependency concept can be successfully applied to problem gambling. This application is problematic due to the stigma. The stress-coping model is proposed as a more empowering perspective, framing these behaviours as adaptive strategies. | |  |
| **Coffman & Swank, 2020**  ***USA*** |  | |  |
| Type | Theoretical paper | |  |
| Study Aim | To explain the association between attachment styles and SUD within family systems. | |  |
| Population | N/A | |  |
| Method | Narrative synthesis of existing literature. | |  |
| Co-dependency definition | A dysfunctional learned behaviour pattern influenced by insecure attachment styles. | |  |
| Framework | Attachment theory; Family system theory. | |  |
| Findings | Insecure attachment styles are predictors of poor emotion regulation and interpersonal communication problems, which in turn may lead to codependent behaviours. SUD in families significantly impacts attachment systems, leading to increased co-dependency. | |  |
| **Kolenova et al., 2023**  ***Russia*** |  | |  |
| Type | Theoretical paper. | |  |
| Study Aim | To analytically review scientific approaches to the study of the features of psychological markers of co-dependency.. | |  |
| Population | N/A | |  |
| Method | Theoretical review. | |  |
| Co-dependency definition | A non-chemical addiction caused by a change in value-semantic constructs and a lack of necessary competencies, formed under the influence of negative experience of dysfunctional relationships. | |  |
| Framework | Biopsychosocial perspective; Cognitive/personality psychology. | |  |
| Findings | Psychological markers of codependent behaviour are manifested through a learned set of behavioural patterns, adaptation disorders, and associations with various PD. Co-dependency is associated to anxiety, depression and stress and has high comorbidity with PDs. | |  |
| **Liverano et al., 2020**  ***Italy*** |  | |  |
| Type | Theoretical paper | |  |
| Study Aim | To describe the etiopathogenetic origins of love addiction. To consider the connection between attachment and love addiction. To introduce a protocol for working with love addiction using Transactional Analysis. | |  |
| Population | N/A | |  |
| Method | Literature integration and development of a treatment protocol based on Transactional Analysis (TA). | |  |
| Co-dependency definition | The most common type of love addiction. | |  |
| Framework | Attachment theory; Psychodynamic theory; Transactional analysis model. | |  |
| Findings | Co-dependency is characterised by low self-esteem, insecurity, and a need to hold onto a partner to fulfil unmet needs. Co-dependents tolerate mistreatment and assume a caregiver role. | |  |
| **Shishkova & Bocharov, 2022**  ***Russia*** |  | |  |
| Type | Theoretical review | |  |
| Study Aim | To identify the barriers and benefits of applying the burnout concept rather than co-dependency in the context of the relationships between addicts and their relatives. | |  |
| Population | N/A | |  |
| Method | N/A | |  |
| Co-dependency definition | A phenomenon rooted in the stigma associated with traditional female roles in families dealing with addiction. The behaviours traditionally associated with co-dependency are instead conceptualised as a result of stress and burnout | |  |
| Framework | Sociocultural perspective; Burnout/Stress-coping perspective. | |  |
| Findings | Co-dependency frames caregiving behaviours as dysfunctional. The stress-coping model can be employed instead to emphasise adaptive strategies. | |  |
| **Weiss, 2019**  ***USA*** |  | |  |
| Type | Expert opinion with theoretical and quantitative elements | |  |
| Study Aim | To introduce and evaluate the Prodependence model as an alternative to co-dependency.  To explore whether clinicians view prodependence as a more welcoming and potentially more effective paradigm. | |  |
| Population | Clinicians treating loved ones of sex addict. Sample size: 64. Age and gender: not reported | |  |
| Method | Informal clinician survey (pre/post educational session) to support conceptual model (Prodependence). Analysed using descriptive survey analysis. Sampling strategy: purposive  Data collection: A survey administered pre and post a presentation on Prodependence assessing familiarity and opinions on the co-dependency and Prodependence models. Data analysis: descriptive survey analysis | |  |
| Co-dependency definition | A deficit-based, trauma-informed model, where caring for others is seen as dysfunctional behaviour. | |  |
| Framework | Attachment theory; Stress-coping perspective. | |  |
| Findings | The co-dependency model implies that caring for others is inherently dysfunctional. In contrast, Prodependence is introduced and welcomed as an alternative strength-based, attachment-focused model. This model frames the behaviour of loved ones who continue to support addicts as normative and rational responses to a relational crisis. | |  |
| **Winter, 2019**  ***Sweden*** |  | |  |
| Type | Theoretical with qualitative elements | |  |
| Study Aim | To explore how co-dependency knowledge is produced and communicated within a semi-scientific collaboration involving experts and the public. | |  |
| Population | Participants in the Forum for Research on Drug Dependence network events in Sweden. | |  |
| Method | Sampling strategy: N/A, Data collection: Observations, website materials, and field notes from a public meeting. Data analysis: Content analysis. | |  |
| Co-dependency definition | A construct shaped by societal narratives of victimisation and the brain disease model. | |  |
| Framework | Social constructionism | |  |
| Findings | Co-dependency knowledge was shaped by victimisation narratives and the biological model of addiction. Professionals may have had an agenda to promote the biological model to ensure co-dependency aligns with scientific authority. | |  |

| **Author (year)** | **Clear RQs** | **Data addresses RQ** | **Relevant sampling strategy** | **Representative sampling** | **Appropriate measurements** | **Low risk of non-response bias** | **Appropriate statistical analysis** |
| --- | --- | --- | --- | --- | --- | --- | --- |
| Chang, 2018 | Yes | Yes | Yes | Yes | Yes | No | Yes |
| Eshan & Suneel, 2020 | No | Yes | Yes | Yes | Yes | No | Yes |
| Happ et al., 2023 | Yes | Yes | Yes | No | Yes | No | Yes |
| Kaplan, 2023 | Yes | Yes | Yes | No | Yes | Unclear | Yes |
| Evgin & Sümen, 2022 | Yes | Yes | Yes | No | Yes | Unclear | Yes |
| Karaşar, 2020 | Yes | Yes | Yes | Yes | Yes | Unclear | Yes |
| Kaya et al., 2024 | Yes | Yes | Yes | No | Yes | Unclear | Yes |
| Knapek et al., 2021 | Yes | Yes | Yes | Yes | Yes | Unclear | Yes |
| Knapek et al., 2017 | Yes | Yes | Yes | Yes | Yes | Unclear | Yes |
| Lampis et al., 2017 | Yes | Yes | Yes | No | Yes | Unclear | Yes |
| Rozhnova et al., 2020 | No | Yes | Yes | Unclear | Yes | Unclear | Yes |
| Vederhus et al., 2019 | Yes | Yes | Yes | No | Yes | Unclear | Yes |
| Zielinski et al., 2019 | Yes | Yes | Yes | No | Yes | Unclear | Yes |
| Tunca et al., 2024 | Yes | Yes | Yes | Yes | Yes | Unclear | Yes |
| Hawkins& Hawkins, 2014 | Yes | Yes | Yes | Yes | Yes | No | Yes |
| Atintaş & Tutarel-Kışlak, 2019 | Yes | Yes | Yes | Yes | Yes | Unclear | Yes |
| Bespalov et al., 2024 | Yes | Yes | Yes | Yes | Yes | Unclear | Yes |

**Supplementary File 4. Quality Appraisal**

**Table D1**

*Quantitative evidence (MMAT - Hong et al., 2018)*

**Table D2**

| **Author (year)** | **Clear RQs** | **Data addresses RQs** | **Appropriateness of approach** | **Adequate data collection** | **Adequately derived findings** | **Interpretation of results is substantiated by data** | **Coherence of data & analysis** |
| --- | --- | --- | --- | --- | --- | --- | --- |
| Aristizábal, 2020 | Yes | Yes | Yes | Yes | Yes | Yes | Yes |
| Bacon et al., 2020 | Yes | Yes | Yes | Yes | Yes | Yes | Yes |
| Klimczak& Kiejna, 2018 | Yes | Yes | Yes | Yes | Unclear | Yes | Yes |
| Sobol-Goldberg et al., 2024 | Yes | Yes | Yes | Yes | Yes | Yes | Yes |
| Nordgren et al., 2020 | Yes | Yes | Yes | Yes | Yes | Yes | Yes |
| Winter, 2019 | Yes | Yes | Yes | Yes | Yes | Yes | Yes |

*Qualitative evidence appraisal (MMAT – Hong et al., 201*

**Table D3**

*Textual evidence appraisal (JBI - McArthur et al., 2020)*

| **Author (year)** | **Source identified** | **Source has standing experience** | **Population Focused** | **Logical Argument** | **Literature Reference** | **Incongruence Defended** |
| --- | --- | --- | --- | --- | --- | --- |
| Bacon & Conway, 2023 | Yes | Yes | Yes | Yes | Yes | Yes |
| Weiss, 2019 | Yes | Yes | Yes | Yes | No | No |
| Coffman & Swank, 2021 | Yes | Yes | Yes | Yes | Yes | No |
| Liverano et al., 2023 | Yes | Yes | Yes | Yes | Yes | Yes |
| Kolenova et al., 2023 | Yes | Yes | Yes | Yes | Yes | Yes |
| Shishkova & Bocharov, 2022 | Yes | Yes | Yes | Yes | Yes | Yes |
| Calderwood & Rajesparam, 2014 | Yes | Yes | Yes | Yes | Yes | Yes |

**Supplementary File 5. Narrative Grouping**

**Table E1
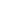
**

*Studies Contributing to Conceptual Categories*

| ***Study*** | ***Sociocultural*** | ***Relational*** | ***Addiction & Pathology*** | ***Developmental*** | ***Psychoanalytic*** | ***Cognitive-Personality*** |
| --- | --- | --- | --- | --- | --- | --- |
| *Calderwood & Rajesparam, 2014* |  | *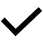* |  |  |  |  |
| *Hawkins& Hawkins, 2014* | *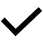* | *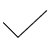* |  |  |  | *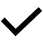* |
| *Knapek et al., 2017* |  | *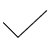* | *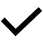* |  |  |  |
| *Lampis et al., 2017* | *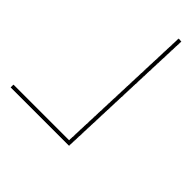* | *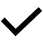* |  |  |  |  |
| *Chang, 2018* | *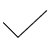* | *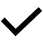* |  | *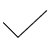* |  |  |
| *Klimczak & Kiejna, 2018* |  | *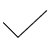* |  | *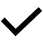* |  | *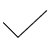* |
| *Atintaş &*  *Tutarel-Kışlak, 2019* | *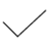* | *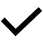* | *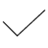* |  |  | *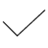* |
| *Vederhus et al., 2019* |  | *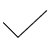* | *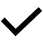* |  |  |  |
| *Weiss, 2019* |  | *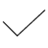* |  | *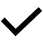* |  |  |
| *Winter, 2019* | *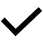* |  |  |  |  |  |
| *Zielinski et al., 2019* |  | *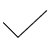* | *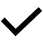* |  |  |  |
| *Aristizábal, 2020* | *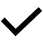* | *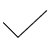* |  |  |  |  |
| *Bacon et al. 2020* |  | *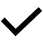* |  | *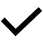* | *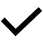* |  |
| *Eshan & Suneel, 2020* |  | *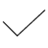* |  |  |  |  |
| *Karaşar, 2020* |  | *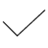* |  |  |  | *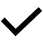* |
| *Nordgren et al., 2020* |  | *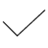* |  |  |  |  |
| *Rozhnova et al., 2020* |  | *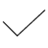* | *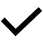* |  |  | *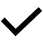* |
| *Coffman & Swank, 2021* |  | *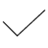* | *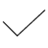* | *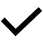* |  |  |
| *Knapek et al., 2021* |  | *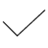* | *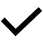* | *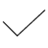* |  | *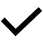* |
| *Evgin & Sümen, 2022* | *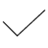* | *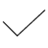* |  | *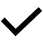* |  |  |
| *Shishkova & Bocharov, 2022* |  | *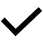* |  |  |  |  |
| *Bacon & Conway, 2023* |  | *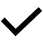* |  |  |  | *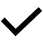* |
| *Happ et al., 2023* |  | *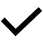* |  |  |  | *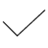* |
| *Kaplan, 2023* | *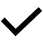* | *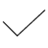* |  |  | *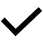* |  |
| *Kaya et al., 2024* |  | *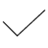* | *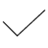* | *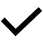* |  | *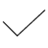* |
| *Kolenova et al., 2023* |  | *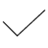* | *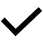* | *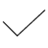* |  | *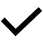* |
| *Liverano et al., 2023* |  | *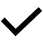* | *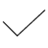* | *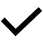* | *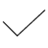* |  |
| *Sobol-Goldberg et al., 2024* | *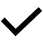* | *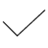* |  |  | *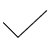* |  |
| *Bespalov et al., 2024* | *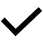* | *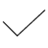* |  | *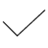* |  | *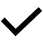* |
| *Tunca et al., 202* |  | *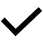* |  | *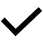* | *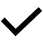* |  |

*Note:* A bold tick (**✓**) indicates a primary or strong conceptual contribution. A faint tick (✓) indicates a secondary or partial contribution. An empty cell indicates no contribution.

**Note. A bold tick (✓) indicates a primary or strong conceptual contribution. A faint tick (✓) indicates a secondary or partial contribution. An empty cell indicates no contribution.**

**Table E2**

*Studies Contributing to Mental Health Outcomes*

**Supplementary File 6. Certainty Assessment**

**Table F1**

*Certainty Assessment*

| **Review Findings** | **Supporting Studies** | **Quality** | **Consistency** | **Relevance** | **Data Richness** | **Confidence Level** |
| --- | --- | --- | --- | --- | --- | --- |
| Co-dependency is conceptualised through developmental frameworks. | 2 qual, 6 quant, 4 conceptual | High | Moderate | High | Moderate/High | Moderate/High |
| Co-dependency is conceptualised through psychoanalytic frameworks. | 2 qual, 2 quant, 1 conceptual | High | Moderate | High | Moderate | Moderate |
| Co-dependency is conceptualised through relational perspectives. | 5 qual, 17 quant, 7 conceptual | High | High | High | High | High |
| Co-dependency is conceptualised through addiction-based and pathology perspectives. | 7 quant, 3 conceptual. | Moderate | Moderate | High | Moderate | Moderate |
| Co-dependency is conceptualised through psychological frameworks. | 1 qual, 8 quant, 2 conceptual | Moderate | Moderate | High | Moderate | Moderate |
| Co-dependency is conceptualised through sociocultural perspectives. | 7 quant, 2 qual, 1 conceptual | High | High | High | Moderate/High | High |
| Co-dependency is linked to identity and self-perception difficulties. | 2 qual, 2 quant | High | High | High | High | High |
| Co-dependency is linked to social functioning and relational difficulties. | 3 quant, 1 qual | High | Moderate | High | Moderate to High | Moderate/High |
| Co-dependency is linked to psychological and emotional difficulties. | 8 quant, 3 qual | High | High | High | High | High |
